# Biostimulation of green microalgae *Chlorella sorokiniana* using nanoparticles of MgO, Ca_10_(PO_4_)_6_(OH)_2,_ and ZnO for increasing biodiesel production

**DOI:** 10.1038/s41598-023-46790-w

**Published:** 2023-11-13

**Authors:** Maryam Faried, Amany Khalifa, Mohamed Samer, Yasser A. Attia, Mohamed A. Moselhy, Ahmed El-Hussein, Rania S. Yousef, Khaled Abdelbary, Essam M. Abdelsalam

**Affiliations:** 1 Department of Agricultural Engineering, Faculty of Agriculture, Cairo University, Giza, Egypt; 2 Department of Laser Applications in Metrology, Photochemistry, and Agriculture, National Institute of Laser Enhanced Sciences, Cairo University, Giza, Egypt; 3 Nanophotonic Research Lab (NRL), Physics Department, The American University in Cairo (AUC), New Cairo, Egypt; 4 Department of Microbiology, Faculty of Agriculture, Cairo University, Giza, Egypt; 5 Faculty of Science, Galala University, Suez, Egypt; 6 Department of Biochemistry, Faculty of Agriculture, Cairo University, Giza, Egypt

**Keywords:** Microbiology, Biogeochemistry, Bioenergy, Lipids, Energy

## Abstract

Microalgae have the potential to become the primary source of biodiesel, catering to a wide range of essential applications such as transportation. This would allow for a significant reduction in dependence on conventional petroleum diesel. This study investigates the effect of biostimulation techniques utilizing nanoparticles of Magnesium oxide MgO, Calcium hydroxyapatite Ca_10_(PO_4_)_6_(OH)_2_, and Zinc oxide ZnO to enhance the biodiesel production of *Chlorella sorokiniana*. By enhancing cell activity, these nanoparticles have demonstrated the ability to improve oil production and subsequently increase biodiesel production. Experimentally, each nanomaterial was introduced at a concentration of 15 mg L^−1^. The results have shown that MgO nanoparticles yielded the highest biodiesel production, with a recorded yield of 61.5 mg L^−1^. Hydroxyapatite nanoparticles, on the other hand, facilitated lipid accumulation. ZnO nanoparticles showcased a multifaceted advantage by enhancing both growth and lipid content. Thus, it is suggested that these nanoparticles can be used effectively to increase the lipid content of microalgae. These findings highlight the potential of biostimulation strategies utilizing MgO, hydroxyapatite, and zinc oxide nanoparticles to bolster biodiesel production.

## Introduction

Climate change has severe consequences on Earth and all human-related activities. The development of efficient and economical renewable biofuels became an essential need rather than a research fantasy^1^. It seems feasible that global climate change, greenhouse emission effects, depleting freshwater resources in some regions, growth in human population, and shortages of agricultural land will favor the use of third-generation biofuel production systems such as microalgae.

Biodiesel is one of the potential candidates that can serve as an effective renewable energy source since it is economical and friendly to the environment^2^. In recent years the request for renewable and sustainable sources of energy has become increasingly vital in combating the global energy crisis and mitigating the harmful effects of greenhouse gas emissions, Biodiesel, derived from various plant sources, has emerged as a promising alternative to fossil fuels due to its lower carbon capture footprint and potential for large-scale production^3,4^. Among the potential feedstocks for biodiesel production, microalgae have captured significant attention due to their high oil content and rapid growth rate making them a promising source for sustainable biofuel production. Green microalgae, particularly *Chlorella sorokiniana*, have demonstrated immense potential in biodiesel production due to their ability to accumulate lipid-rich oil droplets within their cells^5–8^. However, to fully exploit their biodiesel production potential, it is necessary to enhance the lipid accumulation in microalgae cells. This is where the concept of biostimulation using nanoparticles comes into play. The manipulation of microalgae using nanoparticles has emerged as a novel approach to stimulate and enhance the growth and lipid content of microalgae biomass for enhanced biodiesel production^9–13^. Nanoparticles possess unique physicochemical properties that allow them to interact at the cellular level, stimulating various biochemical pathways within microalgae. The precise mechanisms by which these nanoparticles enhance microalgal growth and lipid accumulation are not yet fully understood, but it is believed that they modulate key metabolic pathways related to lipid biosynthesis and photosynthesis^13–15^. Nanoparticles of magnesium oxide (MgO), hydroxy apatite [Ca_10_(PO_4_)_6_(OH)_2_], and zinc oxide (ZnO) have shown potential for inducing physiological changes in microalgae and increasing their lipid content. since algae have nutritional requirements such as N, P, K, Ca, Mg, Mn, Fe, B, and Zn^16–19^ However, these nutritional particles have large sizes which require a longer time to be uptaken by the cells which ultimately increases the Hydraulic Retention Time of the microorganisms^20–24^. Thus, the chief aim of the current research is to solve these problems and increase biodiesel production from algal biomass by nanotechnology. This study hypothesizes that nanomaterials are supposed to biostimulate the algal cell and enhance different cellular activities. This is based on the fact that the rate of cellular uptake is inversely proportional to the size of the nutrient particle. As the nutrient materials have scaled down to the nano size, they will be readily taken at a high rate by the cells. Nevertheless, trace metals that serve as cell nutrients in the nanoscale have dual roles; firstly, to decrease the Hydraulic Retention Time (HRT) and the time to achieve the highest oil content and, therefore, the highest biodiesel production^25–31^. Biostimulator involves the modification of the environment to stimulate existing microorganisms to accomplish the target bioprocesses efficiently. This can be carried out by the addition of various forms of nutrients and electron acceptors^32–35^. Understanding the biostimulation potential of these nanoparticles holds great promise for enhancing the economic viability of microalgae-based biodiesel production. Uncovering the underlying mechanisms and optimizing the conditions for biostimulation hence contribute to the sustainable development of renewable biofuel that can alleviate our dependence on non-renewable resources and reduce the environmental impact of conventional fuels^36–39^.

The novelty of this research lies in its focus on utilizing specific nanoparticles MgO, Ca_10_(PO_4_)_6_(OH)_2_, and ZnO) as biostimulants to enhance biodiesel production from *Chlorella sorokiniana* microalgae. While previous studies mostly examined the use of metal oxide nanoparticles as catalysts during the transesterification stage, this study investigates the influence of nanoparticles as nutrients during the microalgae growth stage. By exploring the effects of these nanoparticles on microalgae growth, biomass productivity, lipid accumulation, and the overall quality of biodiesel, this research provides valuable insights into the potential of nanotechnology for improving biodiesel production from algal biomass.

## Results

### Microalgal biomass

**Figure 1:**
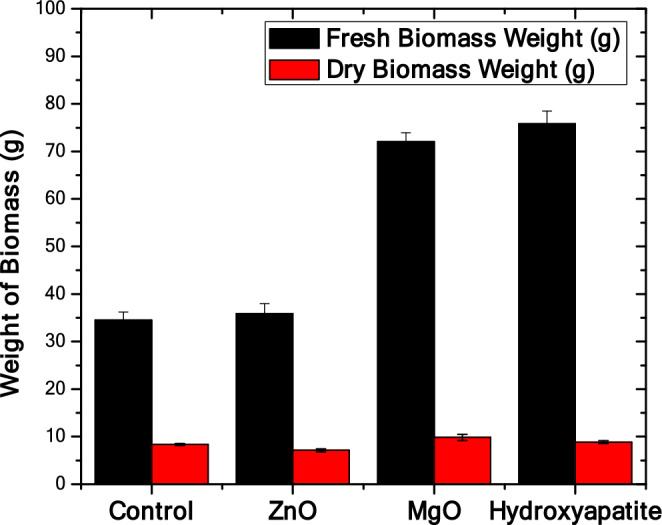
The fresh weight (FW) and the dry weight (DW) of the used biomass in grams for the different treatments (with nanomaterials addition ZnO, MgO, and hydroxyapatite) and the control (without nanomaterials addition).

Figure 1 shows the fresh weight (FW) and the dry weight (DW) of the used biomass in grams for the different treatments (with nanomaterials addition) and the control (without nanomaterials addition).

The usages of different nanomaterials have significantly affected the fresh biomass weight when compared to the control group except for the zinc oxide treated group. Figure 1 shows that the hydroxyapatite-treated group exhibited the highest fresh biomass weight (75.7 g). The zinc oxide treated group didn’t induce much increase in the biomass fresh weight as that increase was statistically insignificant, while magnesium oxide resulted in a significant increase of the fresh biomass (72 g). The increase of fresh biomass that was induced by MgO and hydroxyapatite is statistically significant when compared to both the control and ZnO-treated groups.

Surprisingly, ZnO has led to an insignificant reduction of the biomass dry weight (7.1 g) when compared to the control group (8.3 g) as shown in Fig. 1. However, MgO had increased the biomass dry weight significantly (9.85 g) when compared to the control and ZnO-treated groups. Despite the hydroxy apatite treated group showing an increase in the biomass dry weight as shown in Fig. 1, that increase is statistically insignificant to both control and MgO experimental groups. That increase was significant only when compared to the ZnO-treated group.

### Oil content and moisture

**Figure 2:**
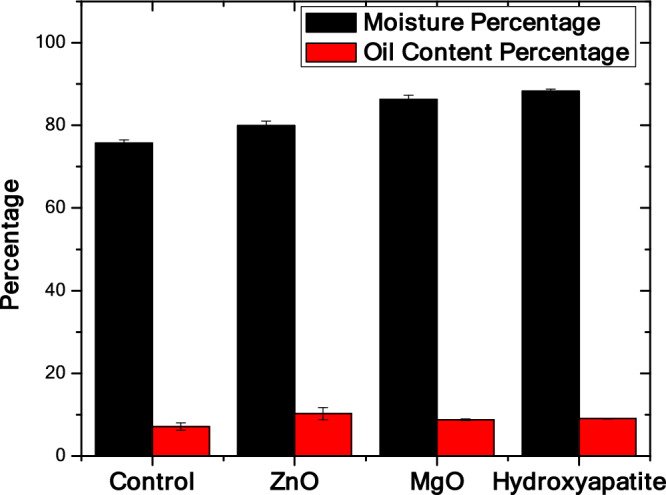
Fisher test of Moisture and oil content percentage for control, ZnO, MgO, and hydroxyapatite experimental groups.

Figure 2 demonstrates both moisture percentage (%) and oil content (%) for the different treatments (with nanomaterials addition) and the control (without nanomaterials addition). All treated groups showed elevated moisture percentages compared to the control group (75.6%). This moisture percentage increase was found to be statistically significant in all levels except the comparison between Hydroxyapatite treated group (88.2%) and the MgO group (86.2%). The lowest significant moisture percentage increase was seen in the ZnO experimental group (80%). ZnO has led to the highest increase in the oil content percentage among any other treated groups (10.23%) when compared to the control group (7.1%) as shown in Fig. 2. Fisher test showed that the only statistically significant increase was the one of the ZnO group when compared to the controls. Despite MgO and hydroxyapatite increasing the oil content percentage (8.8% and 9% respectively), that elevation of the oil content was statistically insignificant.

### Biodiesel yield

The biodiesel production showed a significant increase by using MgO (0.05 mg L^−1^) as shown in Table 1. ZnO has even resulted in an insignificant decrease in biodiesel production. While hydroxyapatite has led to an increase in biodiesel production, that increase is considered to be insignificant when compared to the control group as shown in Table 1. The reduction of biodiesel production that was caused by the usage of ZnO (0.03 mg L^−1^) was found to be statistically significant when compared to the MgO and hydroxyapatite (0.044 mg L^−1^) as shown in Table 1.

**Table 1:** Descriptive results of the biodiesel production (mg L^−1^) by different experimental treated groups, ZnO NPs, MgO NPs, HAP NPs, and control.

	Sample size			Mean	Standard deviation	SE of mean
Control	3			0.0420	1E−3	5.773E−4
ZnO	3			0.0361	0.0051	0.00297
MgO	3			0.0547	0.0059	0.00342
Hydroxyapatite	3			0.0441	0.0063	0.00368

	Fisher test					
	Mean Diff	SEM	t Value	Sig	LCL	UCL
MgO/Control	0.0127	0.00415	3.06883	1	0.00316	0.02229
MgO/ZnO	0.0186	0.00415	4.48618	1	0.00904	0.02816
Hydroxyapatite/MgO	− 0.0105	0.00415	− 2.55452	1	− 0.02015	− 0.00103

### Chemical analyses

#### Fatty acid composition

**Figure 3:**
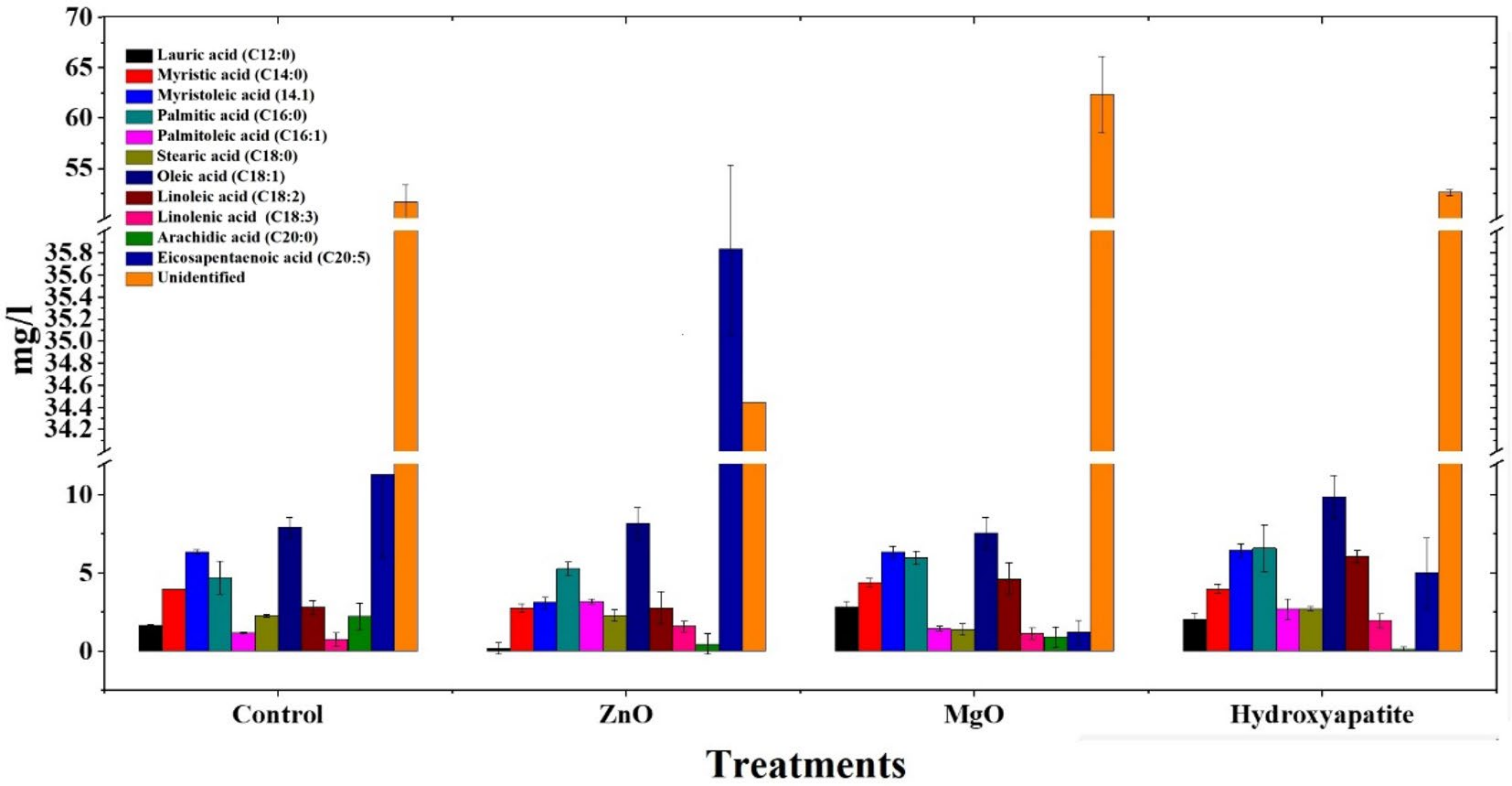
The impact of using ZnO, MgO, and HAP nanomaterials experimental treated groups specific amino acids expression relative to the control group. It shows 11 different fatty acids which have revealed the variable effects of the used nanomaterials on the expression of those fatty acids.

The metabolomics investigations on more than 11 different fatty acids have revealed the variable effects of the used nanomaterials on the expression of those fatty acids as shown in Fig. 3. For instance, ZnO has increased the production of Eicosapentaenoic acid when compared to the control and any other treated group, while it caused the reduction of most of the other examined fatty acids when compared to the controls. The MgO group showed elevated expression of unidentified fatty acids as well as lauric, linoleic, palmitic, and myristic fatty acids when compared to the controls. Eicosapentaenoic and arachidic fatty acids are clearly depressed by the usage of MgO as shown in Fig. 3. The latter behaved the same with the usage of hydroxyapatite, while this experimental group showed increased expression of palmitic, palmitoleic, stearic, and oleic fatty acids as shown in Fig. 3.

#### Morphological changes

**Figure 4:**
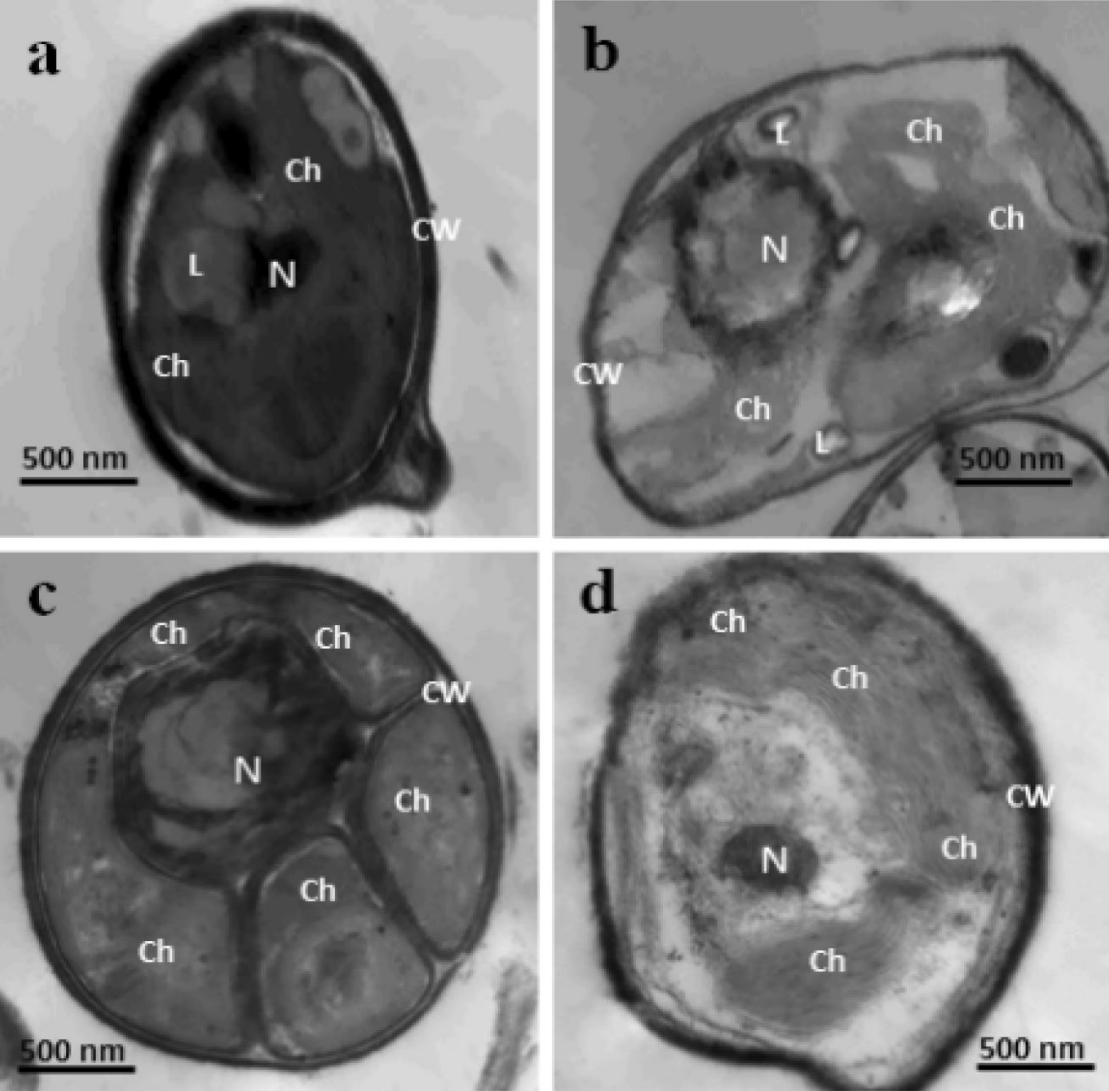
Cell ultrastructure of *Chlorella sorokiniana* grown in BG11 medium supplemented with nanoparticles (NPs); (**a**) Control medium, (**b**) Hydroxyapatite NPs, (**c**) Magnesium oxide NPs, and (**d**) Zinc oxide NPs. Ch, chloroplast; L, lipid droplets; N, nucleus; CW, cell wall.

Based on the obtained results, supplementation of *Chlorella sorokiniana* growth medium with the tested nanoparticles led to cell morphological changes. The most apparent changes were the increase in cell contents, size and the number of chloroplasts as viewed by transmission electron microscope (TEM, Fig. 4). This effect was more pronounced in the presence of magnesium oxide (MgO) and zinc oxide (ZnO) nanoparticles as shown in Fig. 4c,d, respectively.

#### Microalgal count

Microalgal cell counts were determined by the Neubauer counting chamber. The final *Chlorella* cell counts have been affected by the application of the used nanomaterials. The stepwise of magnesium oxide nanoparticles supported the growth of *Chlorella sorokiniana* as indicated by the highest cell counts followed by the effect of the hydroxyapatite nanomaterials compared with the control. Whereas zinc oxide nanoparticles had little inhibitory effect on the microalgal growth as compared to the control (Table 2).

**Table 2:** Chlorella cell counts.

Treatments	Microalgal count log10 cell/ml
Control	6.99 ± 0.17
Zinc oxide (ZnO)	6.54 ± 0.36
Magnesium oxide (MgO)	7.93 ± 0.09
Hydroxyapatite	7.51 ± 0.13

### Results of nanomaterials analysis

**Figure 5:**
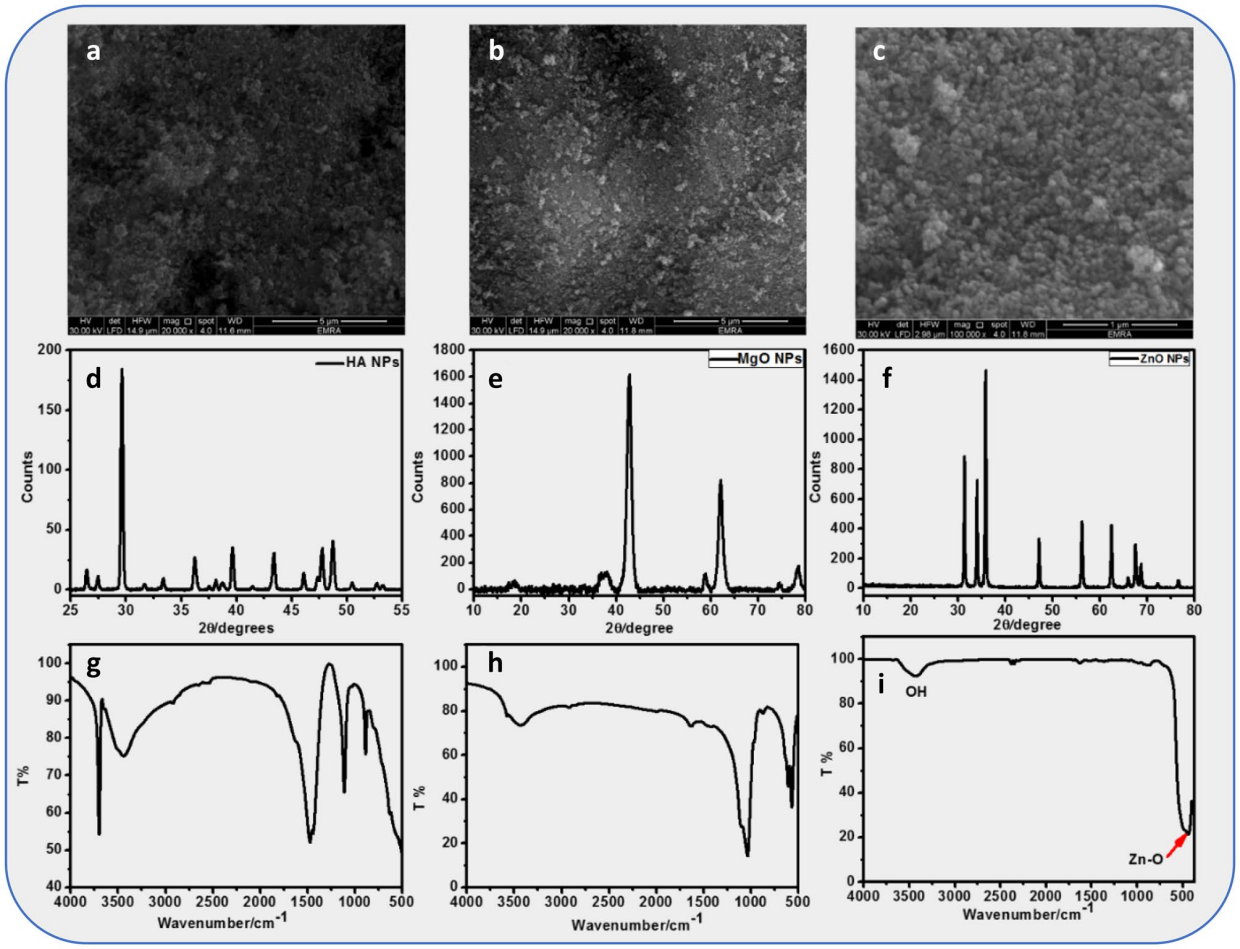
Nanomaterials analysis: (**a**) SEM of HAP NPs, (**b**) SEM of MgO NPs, (**c**) SEM of ZnO NPs, (**d**) XRD of HAP NPs, (**e**) XRD of MgO NPs, (**f**) XRD of ZnO NPs, (**g**) FTIR of HAP NPs, (**h**) FTIR of MgO NPs, and (**I**) FTIR of ZnO NP.

Hydroxyapatite (HAp) exhibits spherical particles with 40 ± 1.8 nm with apparent interconnected porosity (Fig. 5a). The XRD peaks of HAp appearing at 26.2°, 32.2°, and 49.6° (2θ) correspond to the (002), (211), and (123) reflection planes (Fig. 5d). The XRD pattern of the HAp powder obtained is in good agreement with the XRD pattern of a HAp standard available from the Joint Committee on Powder Diffraction Standards (JCPDS; standard number 84-1998). The XRD pattern possesses a strong peak at around 32.2° corresponding to the (211) plane of HAp’s crystalline structure. From the peaks of the XRD diffraction pattern, the prepared HAp samples are in the hexagonal space group (JCPDS 84-1998). The XRD pattern possesses a strong peak at around 32.2° corresponding to the (211) plane of HAp’s crystalline structure. From the peaks of the XRD diffraction pattern, the prepared HAp samples are in the hexagonal space group (JCPDS 84-1998).

The FTIR spectra of HAp samples are shown in Fig. 5g. The IR spectra show the absorption bands at 3696 cm^−1^ and 3441 cm^−1^ which correspond to the stretching mode of the hydroxyl group. The hydroxyl vibration mode is found to be present near 626 cm^−1^. The absorption band appearing in the range 1108 cm^−1^ is due to ν_3_ vibrations of (PO_4_)^−3^. The band at 884 cm^−1^ is due to ν_1_ fundamental mode of (PO_4_)^−3^. The IR spectrum shows an additional absorption band at 3669 cm^−1^ which corresponds to hydroxyl stretching mode associated with surface P–OH groups and the presence of a small amount of CO_3_ can be indicated by the bands appearing at 1440 cm^−1^ showing the formation of carbonate apatite^40,41^.

MgO and ZnO nanoparticles were successfully synthesized by the co-precipitation method using MgCl_2_ and ZnSO_4_ with NaOH as precursors, respectively^42^. The SEM images of MgO and ZnO NPs are depicted in Fig. 5b,c. It is observed that MgO and ZnO NPs have a spherical structure with mean diameters of 31 and 40 nm, respectively. In Fig. 5e,f, it was shown that the XRD pattern of the as-prepared MgO NPs showed major five intense peaks at 2θ values of 36.94° (111), 42.68° (200), 62.4° (220), 74.28° (311), and 78.62° (222), which reveals the formation of the polycrystalline cubic structure of MgO nanoparticles. The XRD pattern of the as-prepared ZnO NPs shown in Fig. 5f observes the presence of wurtzite ZnO due to the presence of three distinct features: the first at 2θ = 36.252° is owing to (101) reflection of planes and 2θ = 34.440° and 56.555° are due to (200) and (011) reflection of planes, respectively. The diffraction peaks are quite similar to those of bulk ZnO, which can be indexed as the hexagonal wurtzite structure ZnO and diffraction data were in agreement with the JCPDS card for ZnO (JCPDS 36-1451)^43^.

FTIR spectrum of MgO nanoparticles prepared using the combustion process is shown in Fig. 5h. It shows the stretching vibration mode ~ 566–870 cm^–1^ indicating Mg–O–Mg bonds^44^.

The distinct band is observed in the wave number ~ 1630 cm^**−**1^, denoting the bending vibration of the surface hydroxyl group. Broadband is observed ~ 3429 cm^**−**1^ due to O–H stretching vibration of a water molecule. The broad peak in the range 3300–3600 cm^**−**1^ showed the formation of MgO structure. The FTIR spectrum of ZnO NPs was recorded in the range 390–4000 cm^−1^, and it is given in Fig. 5i. A significant vibration band ranging from 400 to 500 cm^−1^ is assigned to the characteristic stretching mode of the Zn–O bond. The broad peak 3434 cm^−1^ (stretching) indicates the presence of hydroxyl groups of absorbed H_2_O molecules.

## Discussion

Algal biomass was able to produce renewable biofuels via the diverse bioconversion routes. Algae have been seen as a potential biofuel source due to their high algal reproduction rate with their ability to accumulate high contents of high-energy lipids as of their high photosynthetic activities^45^. Moreover, Algae are characterized by possessing an uncomplicated life cycle with high availability of scaling up production that could fix GHGs and release oxygen into the environment^1,46^. Breaking down algal cell structural contents results in the release of high oil yields^47^ which in turn could be converted into a vast range of biofuels^1^. Light is a dominant factor that affects the ability of algae in accumulating lipids and hence came the idea of enhancing lipid accumulation of algae by photobiostimulation^48,49^. Biodiesel is the present and future alternative to non-renewable fuel sources that can meet the increasing world’s fuel demands, especially after the unrest situation in Ukraine and Europe. Biodiesel doesn’t need any engine changes since it exhibits combustion properties similar to petrodiesel^50^.

In the current work, the results revealed that magnesium oxide nanoparticles (MgO NPs) delivered the highest biodiesel yield (61.5 mg L^−1^) compared to all other types of nanomaterials including the control group. These results indicate that magnesium has a vital role in growth as magnesium starvation hinders cell division, by diminishing the cell concentration^51^, also it has a vital role in the microalgae cell wall in addition to chlorophyll molecule structures^52^, where magnesium could be stunted in their growth and lipid accumulation in addition biodiesel production^37^.

HA NPs treatment delivered the second highest biodiesel yield, HA is the most thermodynamically stable calcium phosphate mineral; it could be applied effectively in algae processing for biofuel intermediate recovery, due to better heat integration which enhances energy utilization through process stages^53^. In some cases, the trace metals, and chemical additives in the form of nanomaterials should be photoactivated using laser radiation^49^ to get better results.

Biodiesel quality is affected by the ratio of polyunsaturated fatty acids (PUFAs) and saturated fatty acids (SFAs), the results indicate that MgO NPs delivered the highest SFAs and the lowest PUFAs. Oxidation of PUFAs is not favorable as biodiesel is a non-renewable energy source. This is attributed to the fact that PUFAs (have not less than four double bonds) oxidation results in more nitrogen oxide release with a lower thermal efficiency when compared to biodiesel (mainly consisting of saturated fatty acids)^3^. Nonetheless, upon using algae as biodiesel feedstock, we must consider carefully the ratio of SFAs and unsaturated fatty acids (UFAs)^54^. As UFA's high content could lead to glycerides polymerization and hence bad lubrication properties^55^.

Moreover, produced oils with high oleic acid contents render the obtained biofuel to be of good quality in terms of combustion heat, viscosity, ignition quality, oxidative stability, and lubricity. The mentioned factors are controlled by the profile of FAMEs and the oleic methyl ester since the latter could enhance oxidative stability by lowering its melting temperature^56^. *Chlorella* strains were evident to generate significant oleic acid oil contents, proposing their high ability to be a potential feedstock for high-quality biodiesel production^57^.

Sibi et al.^58^ studied the supplementation effect on *Chlorella vulgaris* growth medium with four types of nano metals (copper, lead oxide, magnesium oxide, and zinc oxide nanoparticles) that led to higher growth rate, biomass, cellular pigments, and lipid production than the control hence showing the positive influence of the tested nanoparticles on the microalgal growth. While^59^ found that the addition of iron oxide nanoparticles (IONPs) to the growth medium of two species of microalgae; *Chlorella pyrenoidosa* and *Chlorella sorokiniana* showed a contradicting effect. IONPs supplementation at a rate of 20 mgL^−1^ resulted in improvement in biomass and lipid production during the cultivation of *C. pyrenoidosa*. Contrariwise, the IONPs, at a low quantity of 2 mgL^−1^ revealed toxicity to *C. sorokiniana*. In this context, Wang et al.^60^ tested using magnetic Fe_3_O_4_ nanoparticles to enhance the biomass and total lipid production by *Chlorella* sp. UJ-3. The results revealed that the algal biomass increased significantly when 20 mg L^−1^ of Fe_3_O_4_ NPs were added to the growth medium, while the highest total lipid content of algal biomass was attained when Fe_3_O_4_ NPs were supplemented at a high concentration of 100 mg L^−1^. In general^61^, concluded that Fe NPs could be appropriate to produce biomass, and other nanoparticles such as Mg, Zn, and Pb are suitable for the stimulation of lipids production, while only Mg NP is proper for the induction of carbohydrates. Thus, the nanoparticles inducing a higher accumulation of lipids would be useful for biodiesel production from algal biomass in large-scale applications.

Nanomaterials displayed stimulating effects on the algae throughout the start-up of the experiment ranging from the Hydraulic Retention Time (HRT) and stretching during the whole experimental period. Furthermore, supplementing nanomaterials reduced the lag phase and the needed period to obtain the greatest oil and biodiesel yields which is equivalent to the production peak. Largely, our findings match with those that were reported in the literature and were conducted on different microorganisms to produce biofuels^31–35,39^.

In the present study, TEM micrographs confirmed that the addition of nanoparticles to BG11 medium, especially MgO NPs increased the number of chloroplasts. Thus, this treatment attained the highest biodiesel yield. In a study conducted by Fu£íková and Lewis^62^, they found that the mature *Chlorella* cells have multiple chloroplasts, whereas the younger cells contain a single chloroplast. Moreover, the existence of multiple chloroplasts in *Chlorella* cells supports the production of larger cell sizes due to the simultaneous accumulation of lipids in storage vacuoles and photosynthetic carbon fixation^63^.

## Conclusions

It can be concluded from this current study that, Supplementing the algae with 15 mg L^−1^ of magnesium oxide nanoparticles (MgO NPs) yields the highest amount of biodiesel. While in case of Supplementing the algae with 15 mg L^−1^ of zinc oxide nanoparticles (ZnO NPs) yields the highest amount of oil. It was found also the control treatment i.e., without nanomaterials addition, yields the lowest amount of oil and biodiesel when compared to any other experimental treatment group. Polyunsaturated fatty acid decreased in magnesium oxide nanoparticles (MgO NPs) treatment on the other hand saturated fatty acid increased which could be improving the biodiesel properties. So, the Current experiments confirmed the positive impact of the used nanoparticles to enhance microalgal biomass production and lipid content. Accordingly, this research can suggest implementing nanotechnology in the algal biodiesel production process by the addition of nanomaterials (nutrients) to algae culture broth, which is an efficacious technique for algae treatment that can be combined with contemporary algal biodiesel production units.

### Future prospectives

It can be suggested that the used metal oxides in this research added to photoactive nanomaterials which have a high tendency to capture and absorb light which is required for the growth of the microalgae hence it will improve the biodiesel production from microalgae.

## Materials and methods

### Experimental Setup

The experimental setup is detailed as follows: designing the photobioreactors array, identifying the suitable nanomaterials, exposing algae to the suitable white LEDs, and choosing the microalgae strains.

### Design of photobioreactors

**Figure 6:**
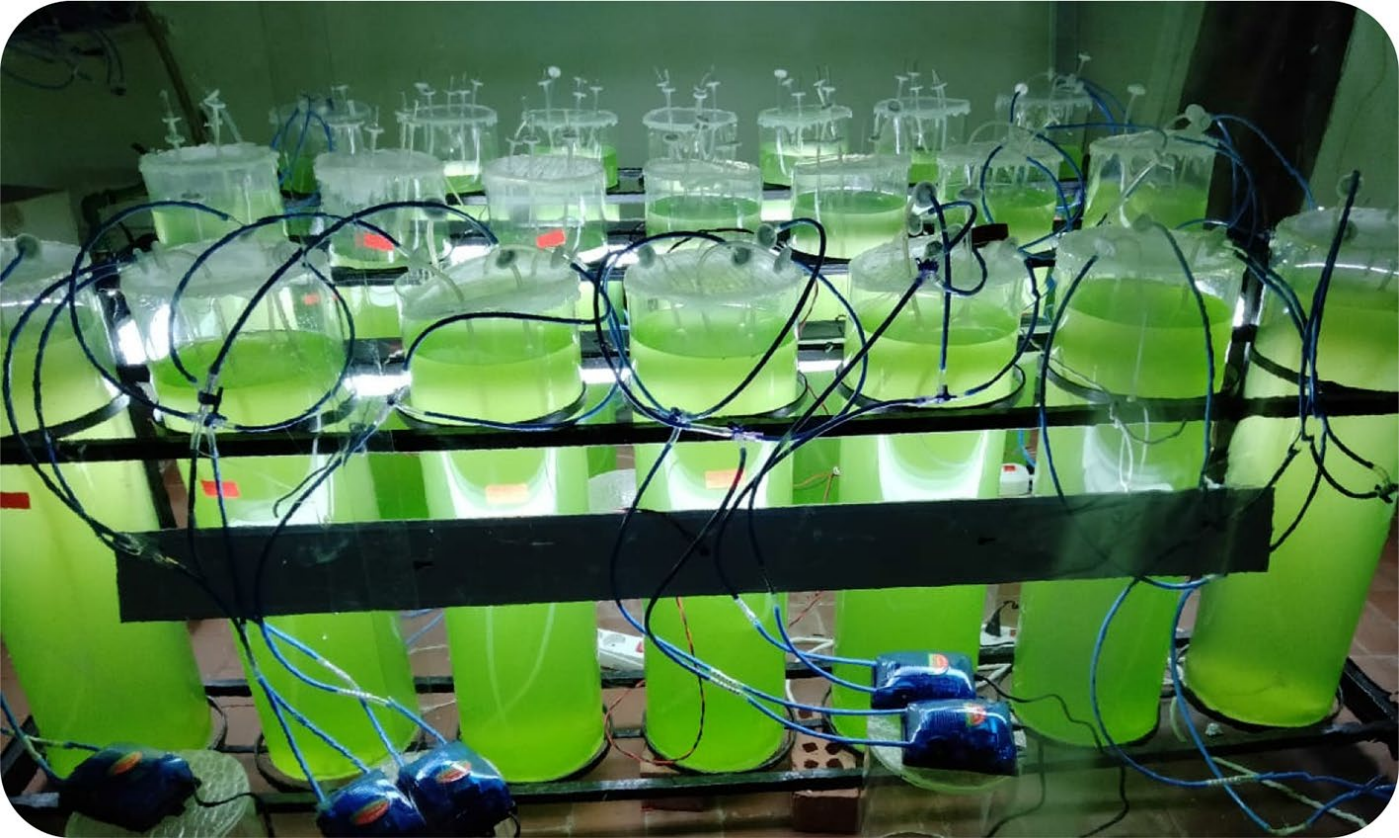
Array of photobioreactors.

Figure 6 shows the photobioreactor home-made array which is mainly adapted from the literature’s lab models and lab scale parameters^64–70^. The photobioreactors were prepared of Poly(methyl methacrylate) (plexiglass) with a diameter of 20 cm, a height of 6,5 cm, and 20 L volume. The photobioreactors were installed in the Biofuel Laboratory, Department of Agricultural Engineering, Faculty of Agriculture, Cairo University.

### Biostimulation using nanomaterials

Algae strains require specific nutrients, which are: nitrogen (N), phosphorus (P), and potassium (K). Additionally, algae require some further nutrients like calcium (Ca), magnesium (Mg), manganese (Mn), iron (Fe), boron (B), and zinc (Zn) that are needed for decent algae growth^25,37^. Therefore, some of the above-mentioned nutrients were prepared in the form of nanomaterials to treat the algal cells. Characterization of the prepared nanomaterials was conducted. The morphology of the prepared nanomaterials (size, shape, crystal structure, etc.) and their optical properties were characterized using X-Ray Diffraction (XRD), Transmission Electron Microscopy (TEM), BET (Brunauer, Emmett, and Teller), Steady-state measurements, Fourier-transform infrared spectroscopy (FTIR) and Specific Surface Area. The following sub-sections elucidate the preparation methods of the different nanomaterials that were used in this study.

### Magnesium oxide nanoparticles

The hydrothermal method was used in the preparation of magnesium oxide (MgO) nanomaterials. The needed chemical in this process is de-ionized water (H_2_O), precursors with suitable quantities such as magnesium chloride (MgCl_2_) or magnesium nitrate (MgNO_3_), and sodium hydroxide (NaOH). The synthetic process mainly relies on the hydroxide thermal decomposition of the preceded hydroxide precipitation. MgO nanoparticles were obtained by precipitating magnesium salt at a coordinated temperature via the usage of 2 mol L^−1^ caustic soda. Stirring vigorously has been applied throughout the synthesis process. After filtration and washing twice of the solid phase, hydrothermal treatment was applied to the samples using the Chandran method to have eventually MgO nanomaterials^36^.

### Calcium phosphate nanoparticles

Calcium phosphate, Ca_10_(PO_4_)_6_(OH)_2_ is the main constituent of Hydroxyapatite (HA). We prepared nanocrystals of HA in the current study by the wet deposition method which is illustrated by the following chemical equation:

$${1}0{\text{Ca}}\left( {{\text{OH}}} \right)_{{2}} + {\text{ 6H}}_{{3}} {\text{PO}}_{{4}} \to {\text{Ca}}_{{{1}0}} \left( {{\text{PO}}_{{4}} } \right)_{{6}} \left( {{\text{OH}}} \right)_{{2}} + {\text{18H}}_{{2}} {\text{O}}$$

The reaction temperature, pH, and the addition rate of reactants influence greatly the physical properties (shape, surface area, purity, stability, and size) of the synthesized nanoparticles. Monocrystalline HA nanoparticles could be obtained if the reaction temperature is < 60 °C. This transition temperature is known as the HA monocrystalline nanoparticles formation limit, where polycrystalline HA nanomaterials would be formed above the temperature. An alternative method to get calcium phosphate nanoparticles of size less than 100 nm, stirring a solution of calcium nitrate tetrahydrate (Ca(NO_3_)_2_.4H_2_O) and diammonium phosphate ((NH_4_)_2_HPO_4_) for 24 h and at room temperature as reported by^34^.

### Zinc oxide nanoparticles

A simple precipitation method was used with zinc acetate or zinc sulfate to synthesize zinc oxide nanoparticles. Sodium hydroxide was used as the starting material of the process, where the sample was calcined for two hours at 300 °C. The precipitate after being filtered, washed, and dried at 100 °C in the lab oven was grounded in a mortar^35^.

### Characterization of nanomaterials

Scanning electron microscopy (SEM) images were obtained with a ZEISS FE-SEM ULTRA Plus (equipped with EDX analyzer) microscope with a Philips CM20 microscope, operating at an accelerating voltage of 200 kV. Several drops from the sample dispersion were deposited onto an aluminum pin stub and left to evaporate at room temperature. X-ray diffraction (XRD) measurements were performed using a Philips PW1710 X-ray diffractometer using Cu Ka radiation (k = 1.54186 Ǻ). The XRD patterns were recorded from 20° to 70° 2θ with a step size of 0.020° 2θ and collecting 10 s per step. FT-IR spectra were recorded with a Nicolet 6700 infrared spectrophotometer to determine the specific functional groups present on the surface^71,72^.

### Microalgae and culture media

#### Microalgal strain

The current study used *Chlorella sorokiniana* SAG 211-8k which was gifted from the Marine Toxin laboratory at the Egyptian Agriculture Research Institute. *Chlorella sorokiniana* was chosen as an oleaginous and low oil content strain (19–20%) to study the biostimulation effects on the enhancement of lipid accumulation and consequently improve biodiesel production.

#### Culture medium

(BG11-broth) Blue Green medium (Sigma-Aldrich, Germany) as a colorless medium was used in the current investigation. Standard protocols were followed to synthesize trace metals that are needed for the BG-11 medium^73^.

#### Experimental design

**Figure 7:**
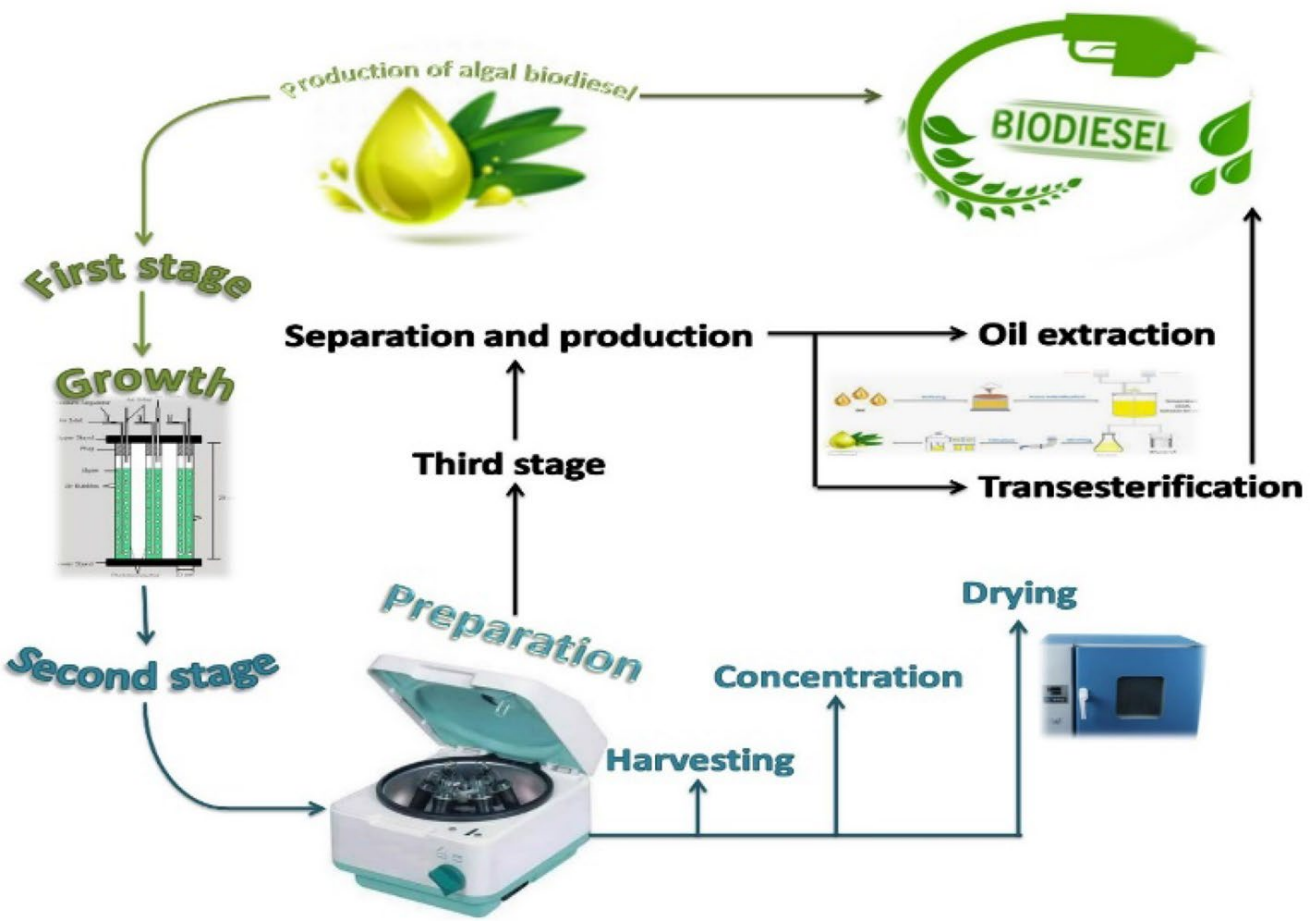
Stages of biodiesel production [MS Office PowerPoint 365].

The biostimulation using nanomaterials and the cultivation of the microalgae processes were done at the Department of Agricultural Engineering, Faculty of Agriculture at Cairo University. The preparation of the used nanomaterials was conducted at the Department of Laser Applications in Metrology, Photochemistry, and Agriculture, the National Institute of Laser Enhanced Sciences (NILES) at Cairo University. Algae produce biodiesel in main three stages as demonstrated in Fig. 7.

To explore the nanomaterial’s influence on algal biodiesel production, 15 mg L^−1^ of Zinc oxide (ZnO), Magnesium oxide (MgO), and Hydroxyapatite was dissolved into a 15 L BG-11 medium. Post biostimulation process, 5 Log10 cell ml^−1^ of microalgal inoculums (about 750 ml) were aseptically inoculated into 15 L BG-11 medium and incubated with constant stirring at 30 ± 5 °C, CO_2_ flow, and a pH of 7.4. The hydraulic retention time (HRT) of the microalgae in the reactor was stretched to reach 21 days. All experimental investigations were done in triplicates. Microalgae biomass was harvested via the usage of centrifugation for 15 min at 5000 rpm. The pellets were washed and dried at 35 °C to have a constant biomass dry weight, which was then grounded to have a fine powder. Worth to mention, that a white LED radiation source of wavelength 380–760 nm was used to irradiate the photobioreactors across the whole hydraulic retention time (HRT) for complete 21 days.

### Measurements

#### Light intensity

A light meter (LX-101, Lutron, Taiwan) was adopted to measure the light intensity of the irradiation source throughout the whole study. The readout unit of the user device is Klux, where (1 Klux = 19.5 μmol m^−2^ s^−1^).

The used white LED source was found to be 1400 lux.

#### Microalgal cell counts

Microalgal cell counts were determined by an improved Neubauer counting chamber with 0.0025 mm^2^ as area and 0.1000 mm as depth. The initial microalgal load was 5.1 Log_10_ cell mL^−1^. Cell density was counted under the microscope by the Neubauer counting chamber, calculated according to the following formula^74^, and then expressed as Log_10_ cell mL^−1^:

1 $${\text{D}} = \frac{{\text{A}}}{{\text{X}}}*{1}0^{{4}}$$

where D = Cell concentration (cell/mL), A = Total number of cells counted (cell), and X = Number of squares.

### Analytical methods

All chemical analytical experiments were measured at the National Research Centre, Egypt.

### Oil extraction

Lipids were extracted from the harvested microalgae of different experimental groups after 21 cultivation days using Soxhlet Reflux Extractor with chloroform/methanol (2/1, v/v) and quantified by the gravimetrical method as early described^25,37^.

### Transesterification

Method for transesterification (using methanol and potassium hydroxide) and characterization of extracted lipids was adopted in the current research^17^.

### Determination of fatty acid composition

The fatty acid composition analysis of the extracts was conducted at Cairo University Research Park (CURP), Faculty of Agriculture using the modified method of^75^, where fatty chains were transmethylated to fatty acid methyl esters (FAMEs). Gas chromatography (Hewlett Packard, USA) has been used to separate FAMEs using Supelco™ SP-2380 (60 m × 0.25 mm × 0.20 μm) column (Sigma-Aldrich, USA). The detector (FID) and the injector temperature were 250 °C. The column temperature was140°C (held for 5 min) and rose to 240 °C, at rate of 4 °C/min, and held at 240 °C for 10 min. The carrier gas was helium at a flow rate of 1.2 mL/min. The sample volume was 1µL (in n-hexane) and injected through a split injector at a splitting ratio of 100:20. FAMEs were identified by comparing their relative and absolute retention times to those authentic standards of FAMEs (Supelco TM 37component FAME mix). The report includes the relative percentage of the total peak after relating the relative and absolute retention times of FAMEs compared to the standards (Supelco™ 37component FAME mix).

The following equation is used to calculate biodiesel content^76^:

2 $${\text{Biodiesel}}\;{\text{content}}\;(\% ) = \left( {\Sigma \;{\text{peak}}\;{\text{areas}}\;{\text{of}}\;{\text{biodiesel}}\;{\text{FAME}}\;{\text{compounds}}\;/\Sigma \;{\text{peak}}\;{\text{areas}}\;{\text{of}}\;{\text{all}}\;{\text{FAME}}\;{\text{compounds}}} \right)*{1}00$$

To obtain the concentration of biodiesel yield (mg/L) from FAME, the following formula has been used^76,77^:

3 $${\text{Biodiesel}}\;{\text{concentration}}\;\left( {{\text{mg}}/{\text{L}}} \right) = \left( {{\text{X}}/{1}00} \right)*{\text{V}}*{\text{D}}*{1}000$$

whereas X: is the percentage of biodiesel; V: is the volume of biodiesel in the sample; D: is the density of the biodiesel.

The FAME chromatogram data for Supelco TM37 external standard, control sample, ZnO, MgO and HA NPs are provided in Supplementary information file.

### Transmission electron microscopy imaging

Microalgal cells were harvested by centrifugation at 5000 × g for 2 min and then washed three times with phosphate buffer (PB). The washed cells were fixed in 2.5% glutaraldehyde for 4 h. The fixed cells were washed three times again using PB, 10 min each. After washing, cells were fixed with 1% osmic acid for 2 h and washed three times with PB for 15 min each run. The fixed cells were dehydrated in ascending series of ethanol (40, 50, 60, 70, 80, 90, 95, and 100%) for 15 min each. The cells were then infiltrated with propylene oxide, then the beam capsule was filled with a resin mixture (Epon 812), and polymerization occurred at 70 °C for 9 h. Ultrathin Sects. (70 nm thick) were cut using an ultramicrotome (RMC Boeckeler, Arizona, USA). The resulting sections were mounted onto 200-mesh copper grids and stained with 2% uranyl acetate and lead citrate for 10 min. The stained sections were examined using a transmission electron microscope (TEM) JEOL -JEM-1200EXII (JEOL Ltd., Tokyo, Japan) at 100 kV.

### Statistical analysis

We have used throughout the current study statistical analysis to investigate the significance of the different experimental observations. We performed a one-way ANOVA and Fisher test (*P* ≤ 0.05) using Origin 8 pro package software (MA, USA).

### Supplementary Information

Supplementary Information 1.

Supplementary Information 2.

Supplementary Information 3.

Supplementary Information 4.

Supplementary Information 5.

Supplementary Information 6.

Supplementary Information 7.

Supplementary Information 8.

Supplementary Information 9.

Supplementary Information 10.

Supplementary Information 11.

Supplementary Information 12.

Supplementary Information 13.

Supplementary Information 14.

## Data Availability

All data generated or analyzed during this study are included in this published article.
