# Author Correction: Biostimulation of green microalgae *Chlorella sorokiniana* using nanoparticles of MgO, Ca_10_(PO_4_)_6_(OH)_2_, and ZnO for increasing biodiesel production

**DOI:** 10.1038/s41598-023-48236-9

**Published:** 2023-12-01

**Authors:** Maryam Faried, Amany Khalifa, Mohamed Samer, Yasser A. Attia, Mohamed A. Moselhy, Ahmed El‑Hussein, Rania S. Yousef, Khaled Abdelbary, Essam M. Abdelsalam

**Affiliations:** 1 Department of Agricultural Engineering, Faculty of Agriculture, Cairo University, Giza, Egypt; 2 Department of Laser Applications in Metrology, Photochemistry, and Agriculture, National Institute of Laser Enhanced Sciences, Cairo University, Giza, Egypt; 3 Nanophotonic Research Lab (NRL), Physics Department, The American University in Cairo (AUC), New Cairo, Egypt; 4 Department of Microbiology, Faculty of Agriculture, Cairo University, Giza, Egypt; 5 Faculty of Science, Galala University, Suez, Egypt; 6 Department of Biochemistry, Faculty of Agriculture, Cairo University, Giza, Egypt

Correction to: *Scientific Reports* https://doi.org/10.1038/s41598-023-46790-w, Published online 13 November 2023

The original version of this Article contained an error in Table 2, where the legend of Figure 4 was incorrectly duplicated in the title of Table 2. The correct title of Table 2 is ‘Chlorella cell counts’.

The original Article has been corrected.

