# Supplementary material for: Biostimulation of green microalgae Chlorella sorokiniana using nanoparticles of MgO, Ca10(PO4)6(OH)2, and ZnO for increasing biodiesel production
Source: Sci Rep. 2023 Nov 13;13:19730. doi: 10.1038/s41598-023-46790-w (PMC10643612; doi:10.1038/s41598-023-46790-w)
Supplement: Supplementary file 1 — Supplementary Information 1. [file 41598_2023_46790_MOESM1_ESM.pdf]

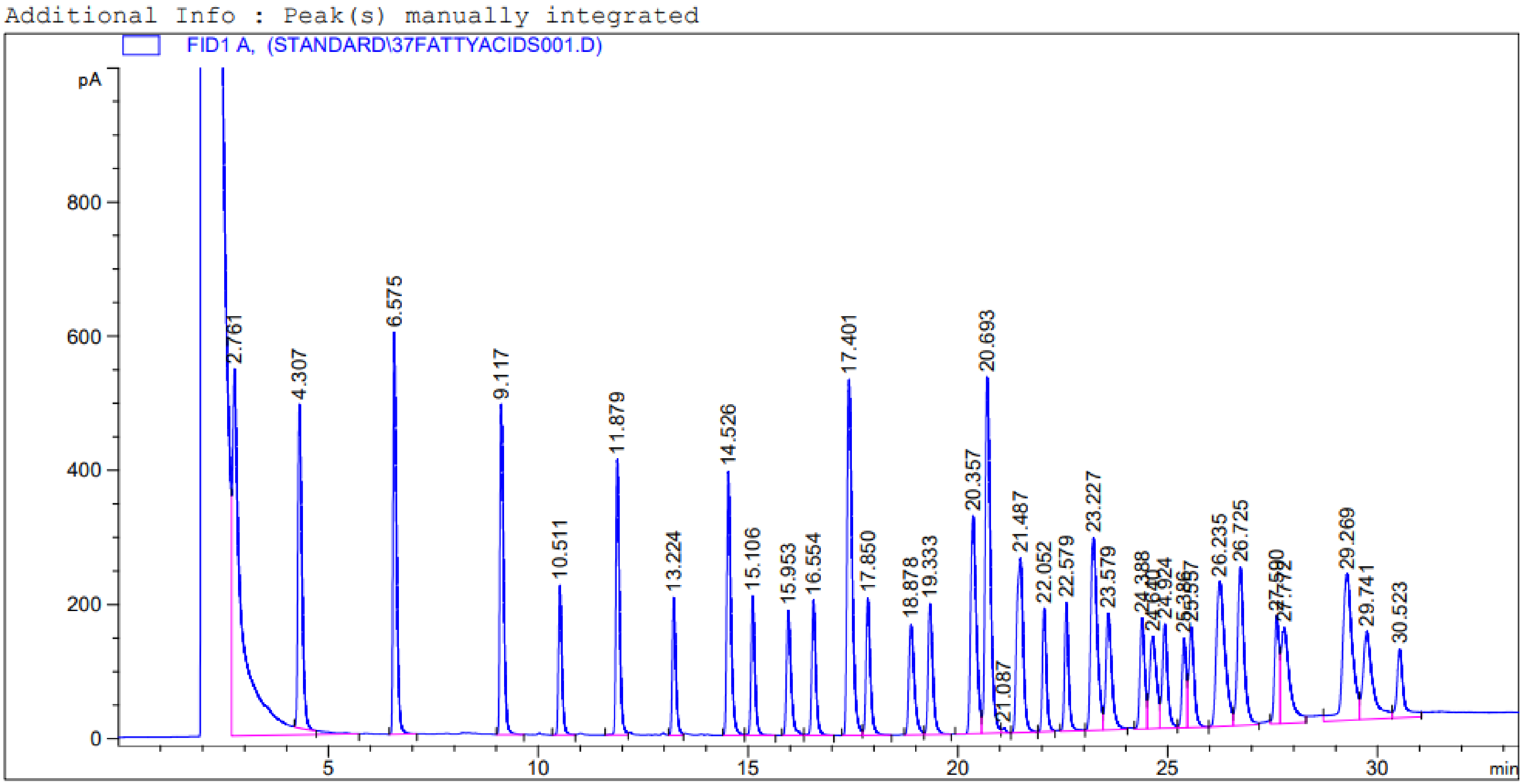

Fig. 1. FAME chromatogram of Supelco TM37 external standard.

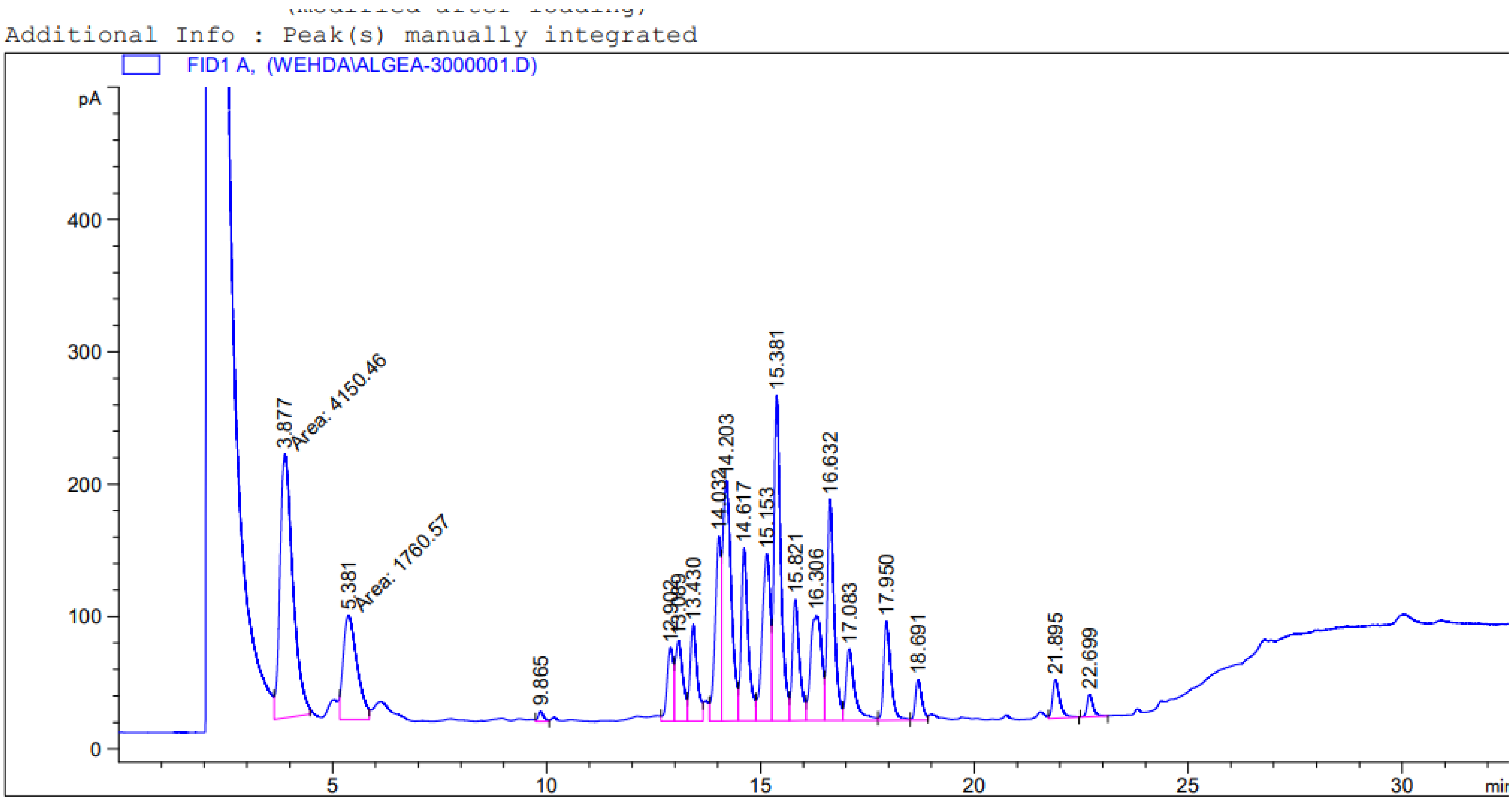

Fig. 2. FAME chromatogram of one replicate of control sample.

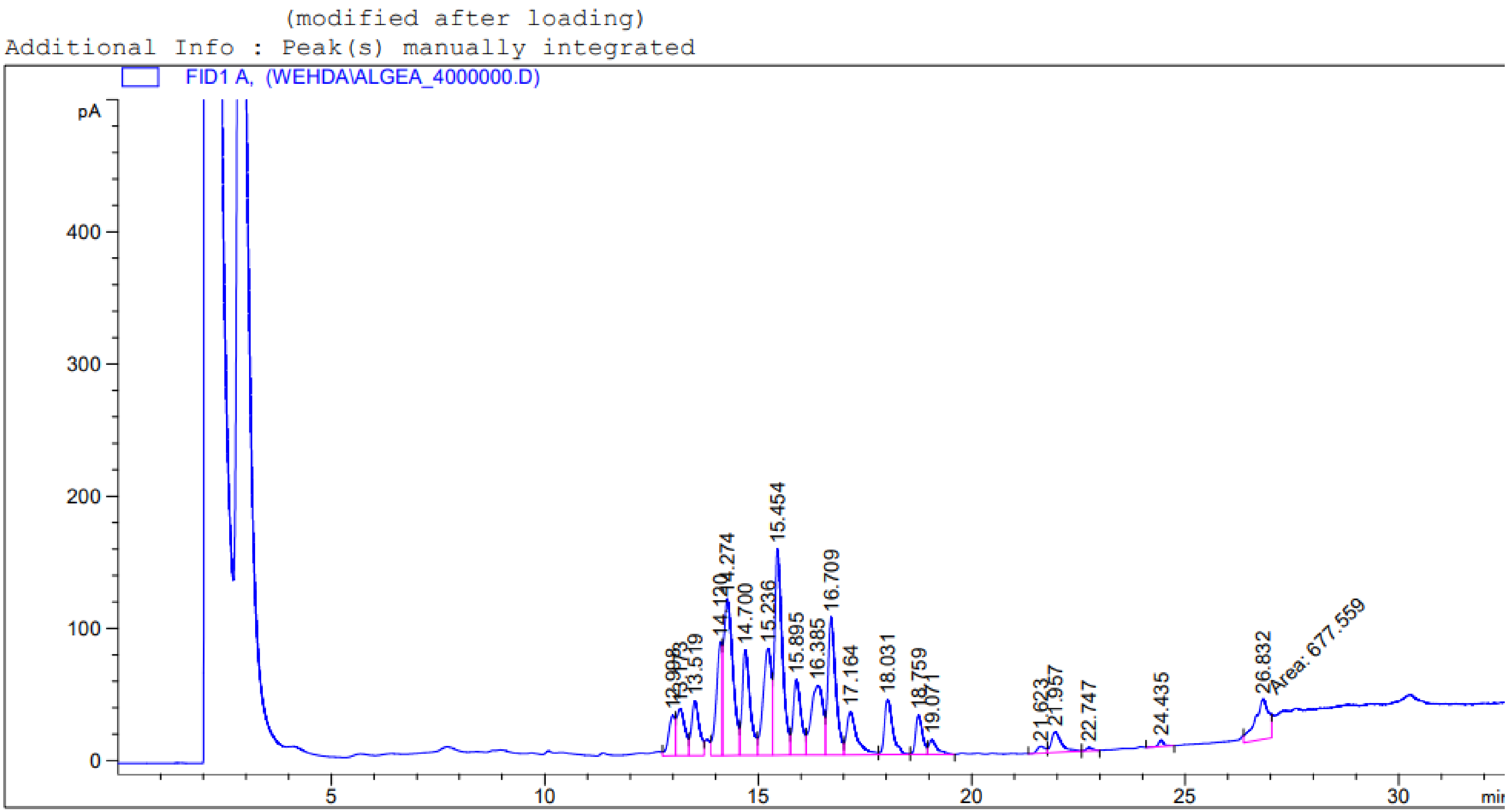

Fig. 3. FAME chromatogram of one replicate of ZnO NPs treatment.

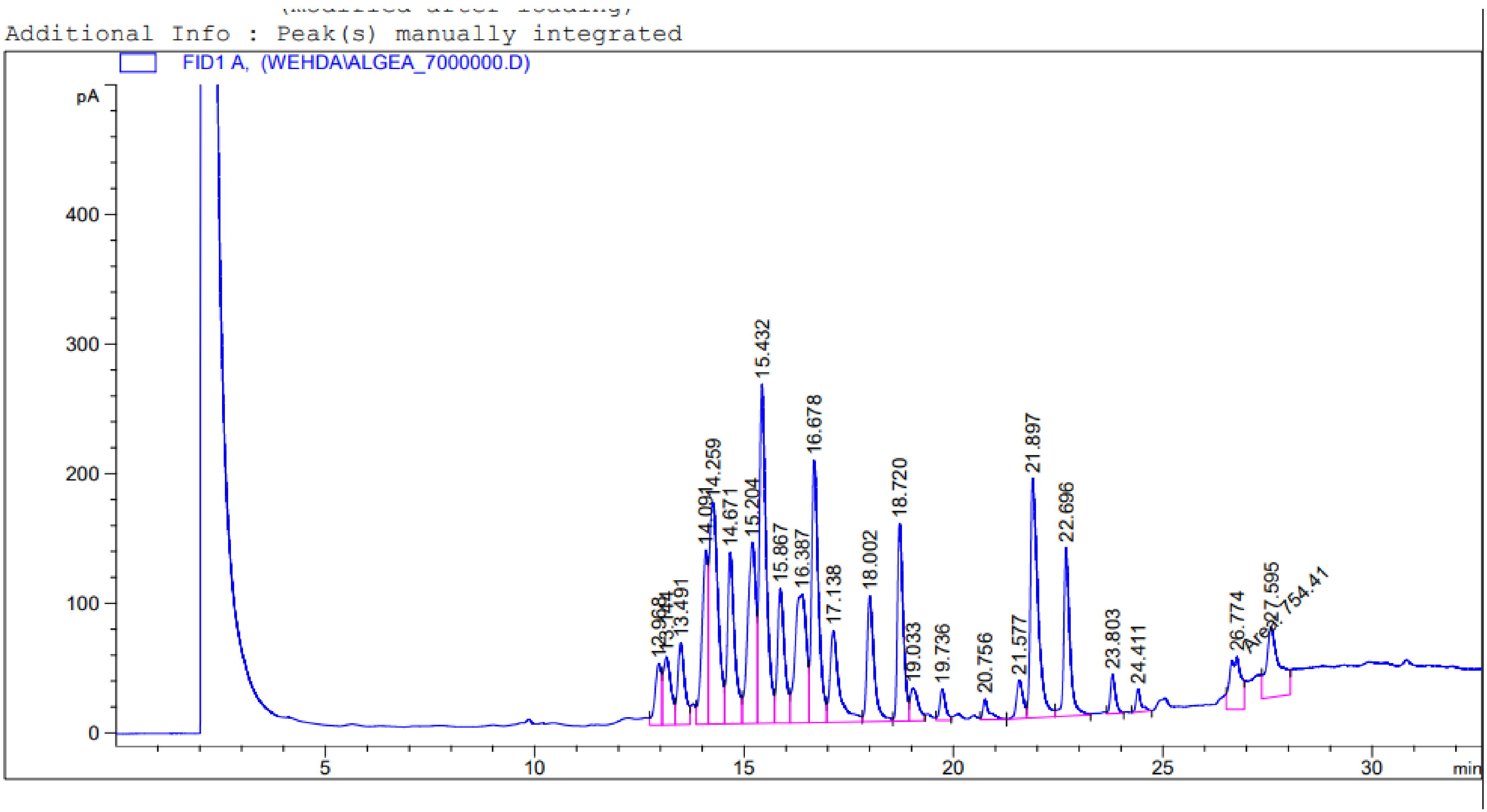

Fig. 4. FAME chromatogram of one replicate of MgO NPs treatment.

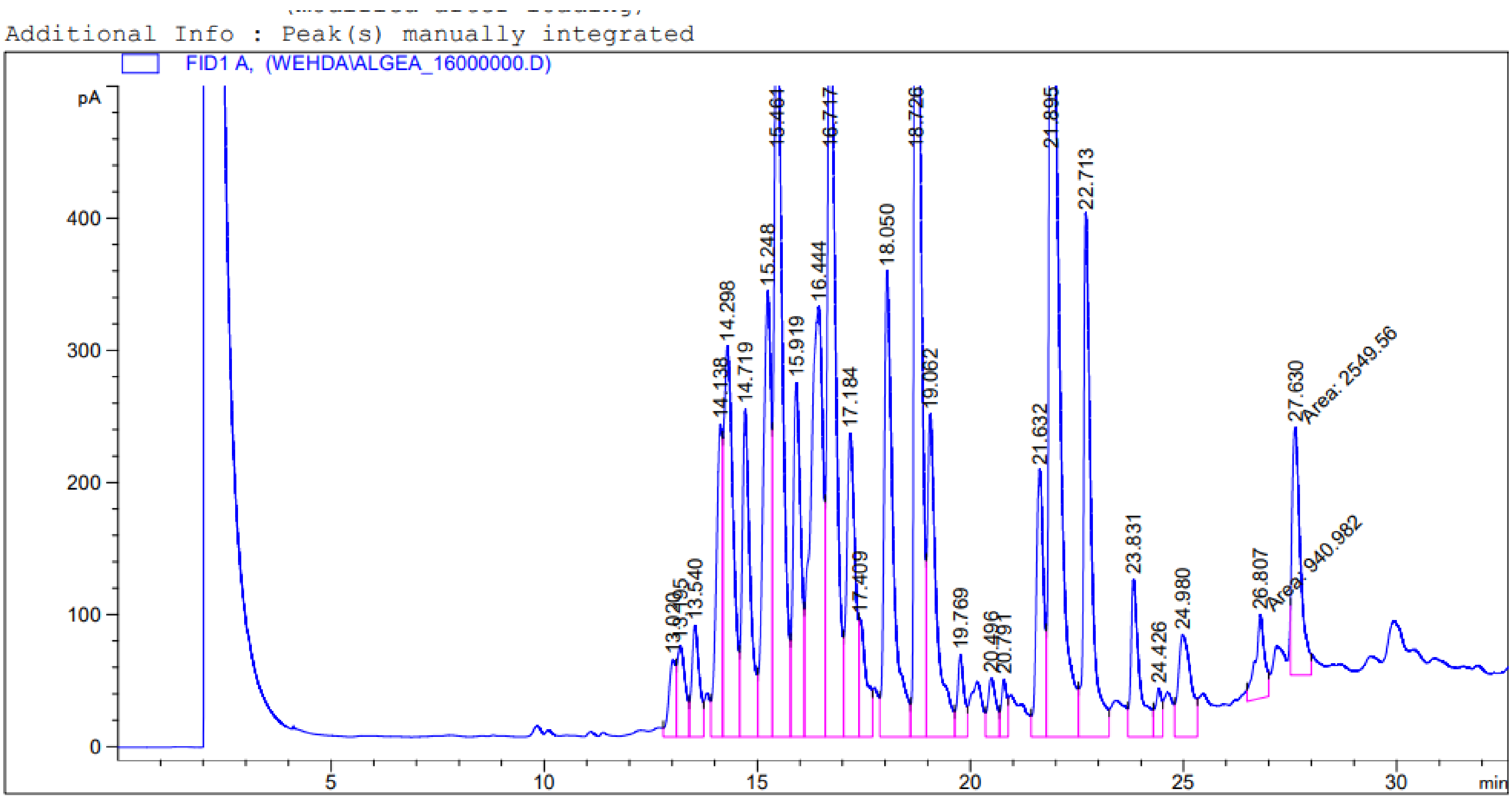

Fig. 5. FAME chromatogram of one replicate of HA NPs treatment.

Table 1. The summary of FAME chromatogram results for experimental samples.

Fatty acids	Control			Zinc oxide (ZnO) (mg/L)			Magnesium oxide (MgO) (mg/L)			Hydroxyapatite (mg/L)		
	**AVE**	**SD**	**SME**	**AVE**	**SD**	**SME**	**AVE**	**SD**	**SME**	**AVE**	**SD**	**SME**
**Lauric acid (C12:0)**	1.662	0.225	0.130	0.170	0.607	0.350	2.827	0.607	0.350	2.007	0.751	0.433
**Myristic acid (C14:0)**	3.973	0.021	0.012	2.767	0.455	0.263	4.383	0.455	0.263	3.967	0.505	0.292
**Myristoleic acid (14.1)**	6.343	0.270	0.156	3.107	0.617	0.356	6.333	0.617	0.356	6.443	0.718	0.415
**Palmitic acid (C16:0)**	4.693	1.851	1.069	5.267	0.732	0.423	5.977	0.732	0.423	6.593	2.596	1.499
**Palmitoleic acid (C16:1)**	1.170	0.111	0.064	3.170	0.254	0.147	1.423	0.254	0.147	2.693	1.137	0.656
**Stearic acid (C18:0)**	2.268	0.135	0.078	2.273	0.607	0.350	1.380	0.607	0.350	2.707	0.239	0.138
**Oleic acid (C18:1)**	7.908	1.095	0.632	8.187	1.735	1.002	7.537	1.735	1.002	9.870	2.370	1.368
**Linoleic acid (C18:2)**	2.783	0.780	0.450	2.767	1.775	1.025	4.607	1.775	1.025	6.050	0.712	0.411
**Linolenic acid (C18:3)**	0.733	0.730	0.421	1.573	0.620	0.358	1.107	0.620	0.358	1.940	0.745	0.430
**Arachidic acid (C20:0)**	2.228	1.465	0.846	0.443	1.139	0.657	0.900	1.139	0.657	0.140	0.242	0.140
**Eicosapentaenoic acid (C20:5)**	11.277	9.190	5.306	35.833	1.341	0.774	1.207	1.341	0.774	4.997	3.908	2.256
**Lignoceric acidC24:0**	2.036	2.035	1.175	–	–	–	–	–	–	–	–	–
**Docosahexanoic acid (C22:6)**	1.257	1.250	0.722	–	–	–	–	–	–	–	–	–
**Unidentified**	51.6670	2.8751	1.6599	34.4433	6.6259	3.8255	62.3200	6.6259	3.8255	52.5933	0.4600	0.2656
