# Supplementary material for: Biostimulation of green microalgae Chlorella sorokiniana using nanoparticles of MgO, Ca10(PO4)6(OH)2, and ZnO for increasing biodiesel production
Source: Sci Rep. 2023 Nov 13;13:19730. doi: 10.1038/s41598-023-46790-w (PMC10643612; doi:10.1038/s41598-023-46790-w)
Supplement: Supplementary file 2 — Supplementary Information 2. [file 41598_2023_46790_MOESM2_ESM.pdf]

Data File C:\CHEM32\1\DATA\WEHDA\ALGEA_ONE000000.D
Sample Name:

===

Acq. Operator : support
Acq. Instrument : Instrument 1   Location : Vial 2
Injection Date : 12/28/2021 11:35:36 AM   Inj : 1
Inj Volume : Manually
Acq. Method : C:\CHEM32\1\METHODS\FAME_NEW.M
Last changed : 12/28/2021 11:28:21 AM by support
Analysis Method : C:\CHEM32\1\METHODS\COOLING.M
Last changed : 9/12/2023 10:41:57 AM
(modified after loading)
Additional Info : Peak(s) manually integrated

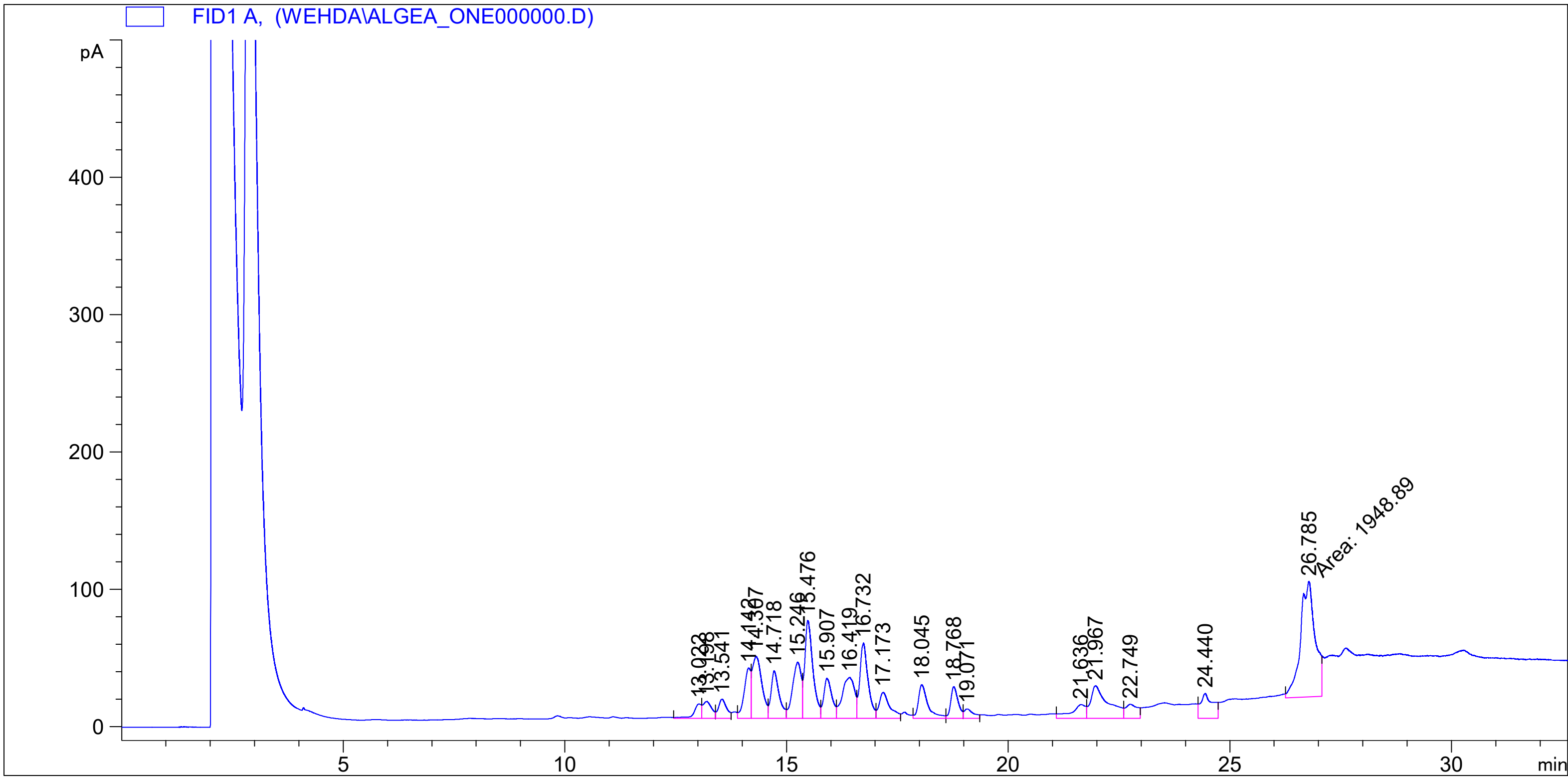

===

Area Percent Report

===

Sorted By : Signal
Multiplier: : 1.0000
Dilution: : 1.0000
Use Multiplier & Dilution Factor with ISTDs

Signal 1: FID1 A,

Peak #	RetTime [min]	Type	Width [min]	Area [pA*s]	Height [pA]	Area %
1	13.022	VV	0.1752	121.71797	10.27835	1.27764
2	13.198	VV	0.1940	168.62053	12.21952	1.76997
3	13.541	VV	0.1930	179.40703	13.77184	1.88319
4	14.142	VV	0.1553	379.50272	36.51559	3.98354
5	14.307	VV	0.1909	683.73401	45.08506	7.17698
6	14.718	VV	0.1912	447.14972	34.51175	4.69362
7	15.246	VV	0.1833	558.09937	40.86896	5.85823
8	15.476	VV	0.1868	916.45813	70.89917	9.61983
9	15.907	VV	0.1734	376.65491	29.03372	3.95365

Peak #	RetTime [min]	Type	Width [min]	Area [pA*s]	Height [pA]	Area %
10	16.419	VV	0.2415	578.68219	29.54816	6.07428
11	16.732	VV	0.1883	704.00433	54.64434	7.38976
12	17.173	VV	0.2334	313.82928	18.83008	3.29419
13	18.045	VV	0.2142	363.69043	24.36371	3.81757
14	18.768	VV	0.1705	267.68100	22.87833	2.80978
15	19.071	VV	0.2001	99.74053	6.76258	1.04695
16	21.636	VV	0.2836	228.71805	9.89662	2.40080
17	21.967	VV	0.3486	648.83813	23.61352	6.81069
18	22.749	VV	0.2441	190.05424	10.16564	1.99495
19	24.440	VB	0.2567	351.28278	17.92091	3.68733
20	26.785	MM	0.3864	1948.89417	84.06372	20.45705
Totals :				9526.75951	595.87157	

===

*** End of Report ***
