# Supplementary material for: Biostimulation of green microalgae Chlorella sorokiniana using nanoparticles of MgO, Ca10(PO4)6(OH)2, and ZnO for increasing biodiesel production
Source: Sci Rep. 2023 Nov 13;13:19730. doi: 10.1038/s41598-023-46790-w (PMC10643612; doi:10.1038/s41598-023-46790-w)
Supplement: Supplementary file 3 — Supplementary Information 3. [file 41598_2023_46790_MOESM3_ESM.pdf]

Data File C:\CHEM32\1\DATA\WEHDA\ALGEA-20000000.D
Sample Name:

===

Acq. Operator : support
Acq. Instrument : Instrument 1 Location : Vial 2
Injection Date : 11/9/2021 1:46:01 PM Inj : 1
Inj Volume : Manually
Acq. Method : C:\CHEM32\1\METHODS\FAME_NEW.M
Last changed : 11/9/2021 1:39:18 PM by support
Analysis Method : C:\CHEM32\1\METHODS\COOLING.M
Last changed : 9/12/2023 10:41:57 AM
(modified after loading)

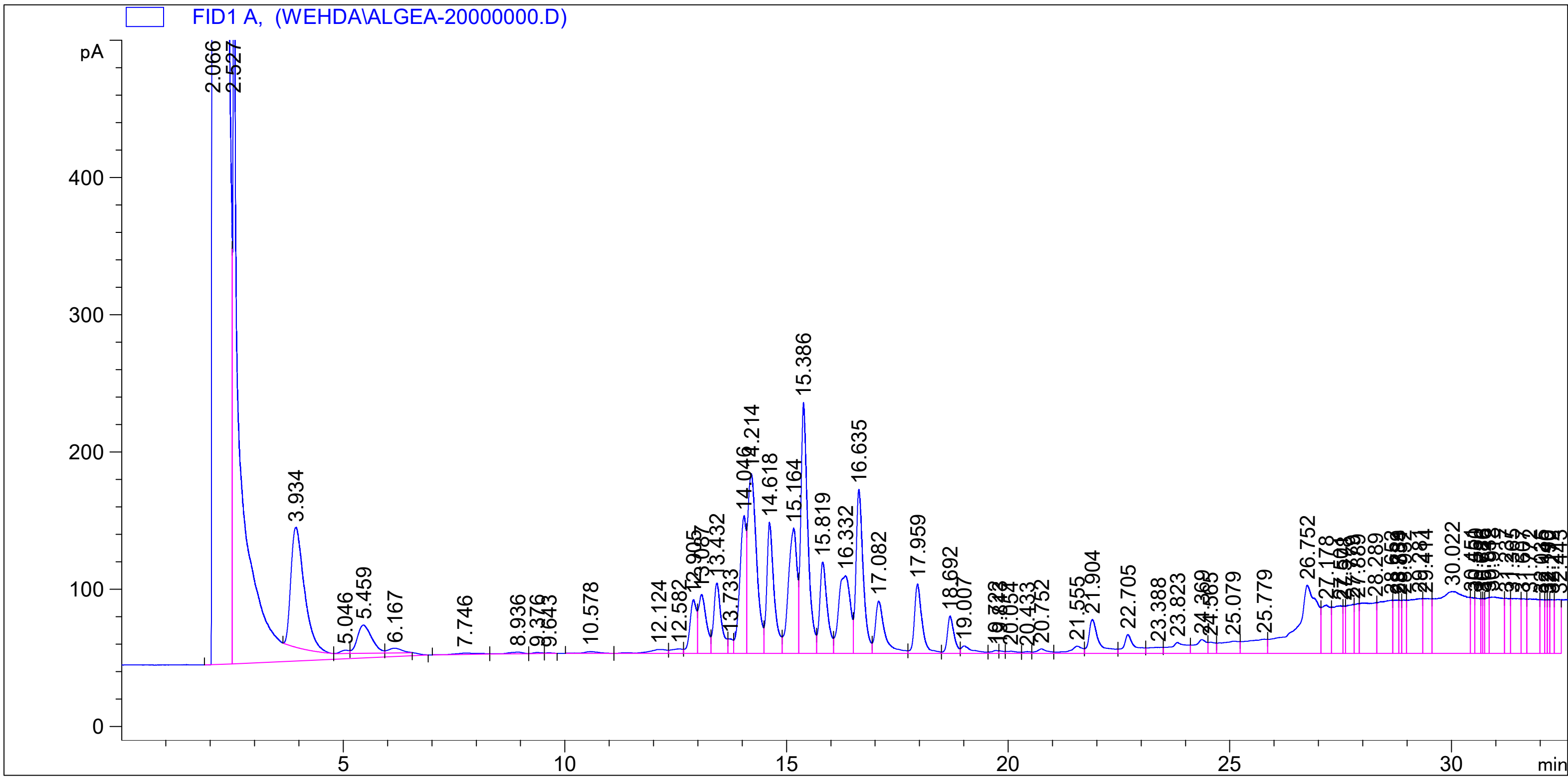

===

Area Percent Report

===

Sorted By : Signal
Multiplier: : 1.0000
Dilution: : 1.0000
Use Multiplier & Dilution Factor with ISTDs

Signal 1: FID1 A,

Peak #	RetTime [min]	Type	Width [min]	Area [pA*s]	Height [pA]	Area %
1	2.066	BV S	0.0359	9.87449e5	4.13588e5	96.10547
2	2.527	VB S	0.1485	7436.07666	834.77460	0.72373
3	3.934	BV X	0.2667	1845.01294	87.25133	0.17957
4	5.046	VV T	0.1688	31.28748	2.37301	0.00305
5	5.459	VV T	0.3025	464.79935	20.50444	0.04524
6	6.167	VB T	0.2508	70.55466	3.39049	0.00687
7	7.746	BV	0.4335	40.11013	1.09459	0.00390
8	8.936	VV	0.3108	31.47580	1.23292	0.00306
9	9.376	VV	0.1674	10.39083	7.65151e-1	0.00101
10	9.643	VB	0.1227	4.97838	4.93273e-1	0.00048

Peak #	RetTime [min]	Type	Width [min]	Area [pA*s]	Height [pA]	Area %
11	10.578	BV	0.2942	31.06839	1.27114	0.00302
12	12.124	VV	0.3532	85.27359	2.89882	0.00830
13	12.582	VV	0.2216	59.39615	3.30593	0.00578
14	12.905	VV	0.1531	402.63211	39.10815	0.03919
15	13.087	VV	0.1762	537.35895	42.87538	0.05230
16	13.432	VV	0.1733	611.31738	51.18695	0.05950
17	13.733	VV	0.0988	81.20462	10.66415	0.00790
18	14.046	VV	0.1438	984.36829	100.04104	0.09581
19	14.214	VV	0.1840	1849.85925	130.81783	0.18004
20	14.618	VV	0.1667	1099.47583	95.24033	0.10701
21	15.164	VV	0.1965	1220.49390	90.96287	0.11879
22	15.386	VV	0.1621	2035.61621	182.50145	0.19812
23	15.819	VV	0.1715	793.90948	66.39162	0.07727
24	16.332	VV	0.2377	1037.38928	56.63841	0.10097
25	16.635	VV	0.1667	1356.89172	119.31770	0.13206
26	17.082	VV	0.2130	569.09430	37.94191	0.05539
27	17.959	VV	0.1734	603.41266	50.51085	0.05873
28	18.692	VV	0.1449	285.90662	27.14401	0.02783
29	19.007	VV	0.2209	96.34565	5.37966	0.00938
30	19.722	VV	0.1338	22.45784	2.11676	0.00219
31	19.813	VV	0.0960	13.40299	1.77767	0.00130
32	20.054	VV	0.2119	26.83656	1.53572	0.00261
33	20.433	VV	0.1532	14.59891	1.25978	0.00142
34	20.752	VV	0.2112	53.59485	3.30544	0.00522
35	21.555	VV	0.2663	101.61278	5.23947	0.00989
36	21.904	VB	0.2186	388.24985	24.54048	0.03779
37	22.705	BV	0.2293	230.67300	13.63910	0.02245
38	23.388	VV	0.2798	99.79247	4.34799	0.00971
39	23.823	VV	0.3413	213.88654	7.81415	0.02082
40	24.369	VV	0.2430	183.26265	9.94270	0.01784
41	24.565	VV	0.1370	89.89082	8.13100	0.00875
42	25.079	VV	0.3535	262.38666	8.85936	0.02554
43	25.779	VV	0.4006	348.95499	10.31639	0.03396
44	26.752	VV	0.4283	1674.77917	49.55241	0.16300
45	27.178	VV	0.1704	481.08008	34.98871	0.04682
46	27.501	VV	0.1871	530.44373	34.54968	0.05163
47	27.578	VV	0.0438	117.43275	34.45552	0.01143
48	27.779	VV	0.1405	409.90530	35.54877	0.03989
49	27.889	VV	0.0928	244.55881	36.19192	0.02380
50	28.289	VV	0.2883	874.72797	36.92962	0.08513
51	28.652	VV	0.2490	808.23944	38.80568	0.07866
52	28.789	VV	0.1121	325.77298	38.72959	0.03171
53	28.834	VV	0.0489	148.50516	38.62361	0.01445
54	28.932	VV	0.0787	249.13477	38.91506	0.02425
55	29.281	VV	0.2577	859.63257	39.85225	0.08367
56	29.414	VV	0.1625	494.38986	39.91058	0.04812
57	30.022	VV	0.5824	2194.93750	45.04840	0.21363
58	30.451	VV	0.0760	236.90466	40.63791	0.02306
59	30.550	VV	0.1029	335.29938	40.35361	0.03263
60	30.686	VV	0.0346	97.70192	39.88095	0.00951
61	30.726	VV	0.0387	104.71293	39.82642	0.01019
62	30.832	VV	0.0786	245.15314	40.48528	0.02386
63	30.918	VV	0.2466	835.23712	40.96987	0.08129
64	31.237	VV	0.0984	325.30728	40.20061	0.03166
65	31.365	VB	0.1741	576.20892	39.98546	0.05608
66	31.607	BV	0.0927	294.95663	39.70839	0.02871
67	31.772	VV	0.2111	696.51416	39.61499	0.06779
68	32.035	VV	0.0829	257.95438	39.15324	0.02511

Peak #	RetTime [min]	Type	Width [min]	Area [pA*s]	Height [pA]	Area %
69	32.140	VV	0.0490	143.79446	39.02156	0.01400
70	32.190	VV	0.0400	120.68709	39.05755	0.01175
71	32.272	VV	0.0786	242.93591	39.06344	0.02364
72	32.443	VV	0.1140	362.68161	39.18849	0.03530

Totals : 1.02746e6 4.16896e5

*** End of Report ***
