# Supplementary material for: Biostimulation of green microalgae Chlorella sorokiniana using nanoparticles of MgO, Ca10(PO4)6(OH)2, and ZnO for increasing biodiesel production
Source: Sci Rep. 2023 Nov 13;13:19730. doi: 10.1038/s41598-023-46790-w (PMC10643612; doi:10.1038/s41598-023-46790-w)
Supplement: Supplementary file 4 — Supplementary Information 4. [file 41598_2023_46790_MOESM4_ESM.pdf]

Data File C:\CHEM32\1\DATA\WEHDA\ALGEA-3000001.D
Sample Name:

===

Acq. Operator : support
Acq. Instrument : Instrument 1 Location : Vial 2
Injection Date : 11/9/2021 1:06:37 PM Inj : 1
Inj Volume : Manually
Acq. Method : C:\CHEM32\1\METHODS\FAME_NEW.M
Last changed : 11/9/2021 1:00:41 PM by support
Analysis Method : C:\CHEM32\1\METHODS\COOLING.M
Last changed : 9/12/2023 10:41:57 AM
(modified after loading)
Additional Info : Peak(s) manually integrated

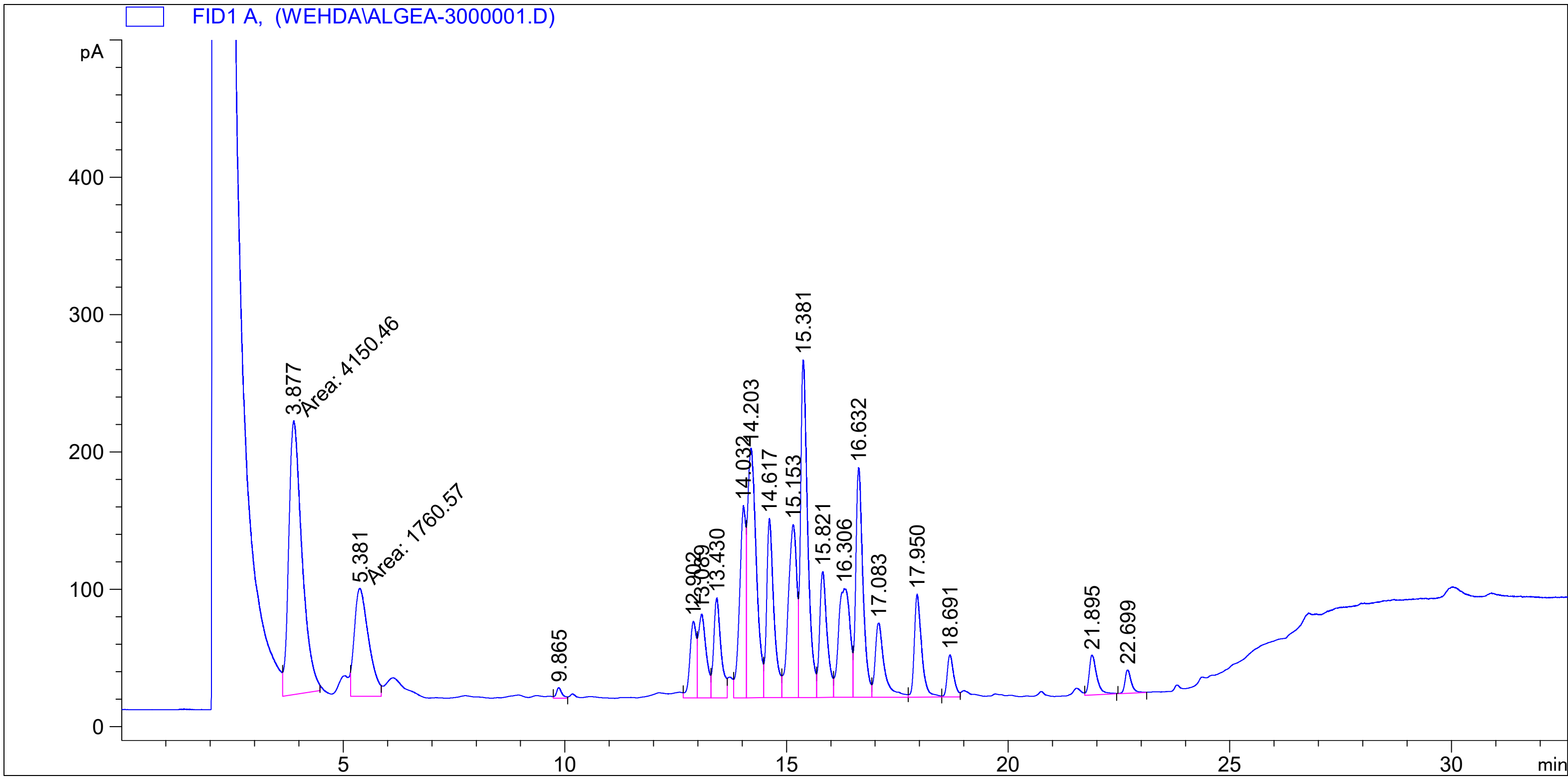

===

Area Percent Report

===

Sorted By : Signal
Multiplier: : 1.0000
Dilution: : 1.0000
Use Multiplier & Dilution Factor with ISTDs

Signal 1: FID1 A,

Peak #	RetTime [min]	Type	Width [min]	Area [pA*s]	Height [pA]	Area %
1	3.877	MM	0.3469	4150.46143	199.38243	16.64921
2	5.381	MM	0.3738	1760.57397	78.49998	7.06239
3	9.865	VV	0.1277	68.30552	7.68364	0.27400
4	12.902	VV	0.1535	561.15240	55.73967	2.25101
5	13.089	VV	0.1813	764.89362	60.59668	3.06830
6	13.430	VV	0.1688	845.35535	72.64088	3.39107
7	14.032	VV	0.1376	1337.85669	139.89697	5.36669
8	14.203	VV	0.1919	2575.10693	181.06110	10.32982
9	14.617	VV	0.1706	1501.85083	130.22028	6.02454

Peak #	RetTime [min]	Type	Width [min]	Area [pA*s]	Height [pA]	Area %
10	15.153	VV	0.1835	1675.15027	125.71490	6.71972
11	15.381	VV	0.1669	2798.87646	245.68626	11.22745
12	15.821	VV	0.1773	1092.21216	91.49334	4.38131
13	16.306	VV	0.2171	1432.25354	79.14767	5.74536
14	16.632	VV	0.1664	1895.67969	166.97919	7.60435
15	17.083	VV	0.2061	774.89575	53.77615	3.10843
16	17.950	VV	0.1677	855.72101	74.65015	3.43265
17	18.691	VV	0.1509	316.59656	30.54015	1.27000
18	21.895	VB	0.1753	349.48267	28.85565	1.40192
19	22.699	BB	0.1504	172.45134	16.98865	0.69177

Totals : 2.49289e4 1839.55375

===

*** End of Report ***
