# Supplementary material for: Biostimulation of green microalgae Chlorella sorokiniana using nanoparticles of MgO, Ca10(PO4)6(OH)2, and ZnO for increasing biodiesel production
Source: Sci Rep. 2023 Nov 13;13:19730. doi: 10.1038/s41598-023-46790-w (PMC10643612; doi:10.1038/s41598-023-46790-w)
Supplement: Supplementary file 5 — Supplementary Information 5. [file 41598_2023_46790_MOESM5_ESM.pdf]

Data File C:\CHEM32\1\DATA\WEHDA\ALGEA_16000000.D
Sample Name:

=====================================================================

Acq. Operator : support
Acq. Instrument : Instrument 1 Location : Vial 2
Injection Date : 12/28/2021 12:15:14 PM Inj : 1
Inj Volume : Manually
Acq. Method : C:\CHEM32\1\METHODS\FAME_NEW.M
Last changed : 12/28/2021 12:08:20 PM by support
Analysis Method : C:\CHEM32\1\METHODS\COOLING.M
Last changed : 9/12/2023 10:41:57 AM
(modified after loading)
Additional Info : Peak(s) manually integrated

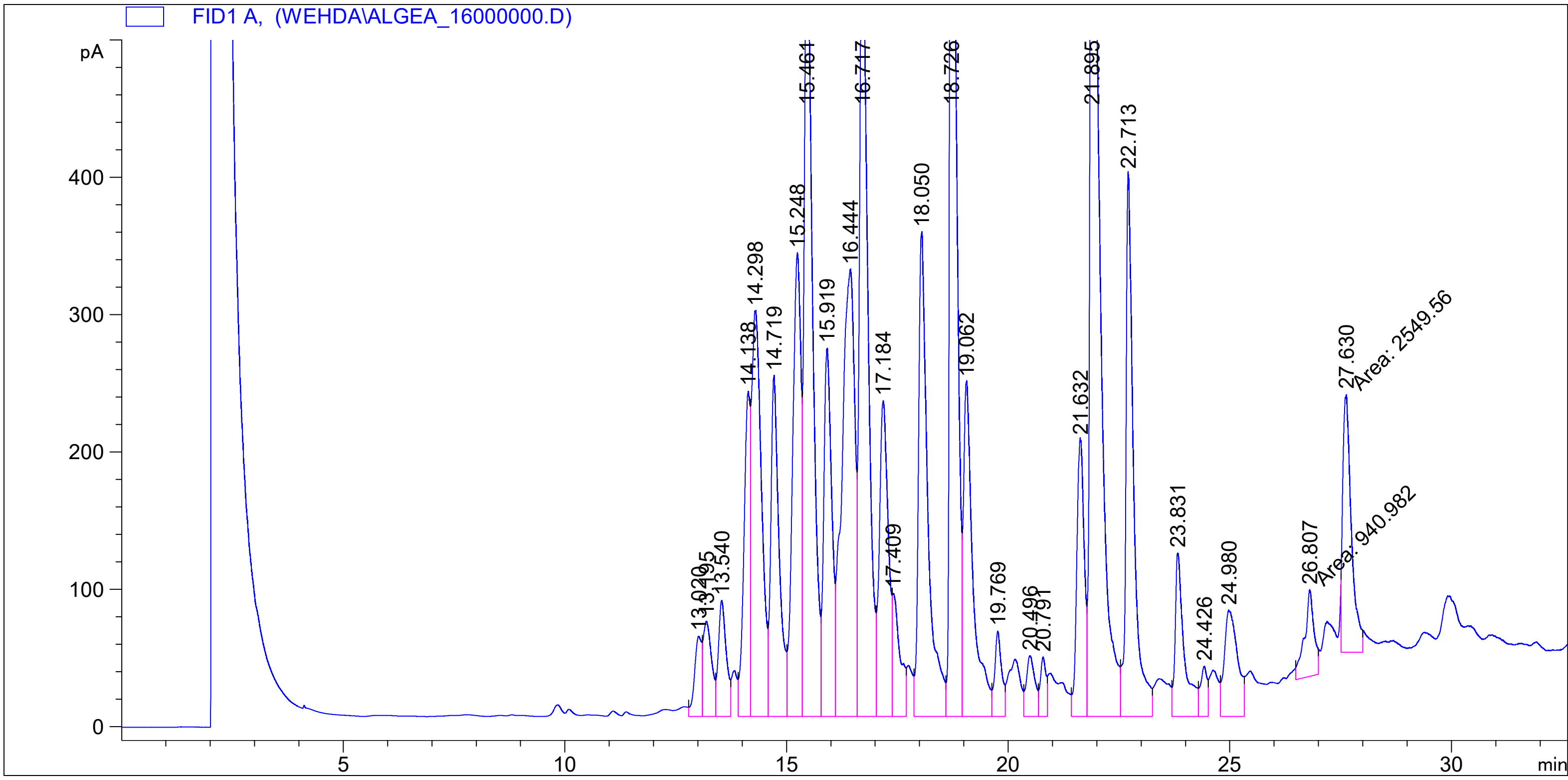


=====================================================================

Area Percent Report

=====================================================================

Sorted By : Signal
Multiplier: : 1.0000
Dilution: : 1.0000
Use Multiplier & Dilution Factor with ISTDs

Signal 1: FID1 A,

| Peak # | RetTime [min] | Type | Width [min] | Area [pA*s] | Height [pA] | Area % |
|---|---|---|---|---|---|---|
| 1 | 13.020 | VV | 0.1558 | 640.89752 | 58.48386 | 0.68796 |
| 2 | 13.195 | VV | 0.1923 | 942.04907 | 69.40849 | 1.01123 |
| 3 | 13.540 | VV | 0.1885 | 1072.97974 | 84.31178 | 1.15177 |
| 4 | 14.138 | VV | 0.1408 | 2289.28247 | 236.70091 | 2.45739 |
| 5 | 14.298 | VV | 0.2266 | 4667.78418 | 295.07880 | 5.01055 |
| 6 | 14.719 | VV | 0.1907 | 3286.72900 | 247.79922 | 3.52808 |
| 7 | 15.248 | VV | 0.1859 | 4448.22021 | 337.13428 | 4.77486 |
| 8 | 15.461 | VV | 0.2010 | 7867.58154 | 584.67596 | 8.44532 |
| 9 | 15.919 | VV | 0.1852 | 3427.15503 | 267.98572 | 3.67882 |

| Peak # | RetTime [min] | Type | Width [min] | Area [pA*s] | Height [pA] | Area % |
|---|---|---|---|---|---|---|
| 10 | 16.444 | VV | 0.2630 | 6606.57129 | 325.17462 | 7.09171 |
| 11 | 16.717 | VV | 0.1955 | 7952.90088 | 589.25153 | 8.53690 |
| 12 | 17.184 | VV | 0.2001 | 3268.40332 | 229.56129 | 3.50841 |
| 13 | 17.409 | VV | 0.1741 | 1088.53345 | 89.37505 | 1.16847 |
| 14 | 18.050 | VV | 0.2114 | 5234.99219 | 352.29681 | 5.61941 |
| 15 | 18.726 | VV | 0.1501 | 8802.54102 | 827.77405 | 9.44893 |
| 16 | 19.062 | VV | 0.2184 | 3718.41846 | 243.20055 | 3.99147 |
| 17 | 19.769 | VV | 0.1593 | 687.66644 | 62.06730 | 0.73816 |
| 18 | 20.496 | VV | 0.1968 | 603.59637 | 44.33887 | 0.64792 |
| 19 | 20.791 | VV | 0.1327 | 394.86496 | 43.15474 | 0.42386 |
| 20 | 21.632 | VV | 0.1811 | 2366.66504 | 201.33510 | 2.54046 |
| 21 | 21.895 | VV | 0.2096 | 1.16969e4 | 768.17950 | 12.55585 |
| 22 | 22.713 | VV | 0.1820 | 4873.36475 | 394.88828 | 5.23123 |
| 23 | 23.831 | VV | 0.1981 | 1712.70764 | 118.84330 | 1.83848 |
| 24 | 24.426 | VV | 0.1493 | 387.44073 | 36.65567 | 0.41589 |
| 25 | 24.980 | VV | 0.2738 | 1630.29346 | 77.36937 | 1.75001 |
| 26 | 26.807 | MM | 0.2495 | 940.98199 | 62.86849 | 1.01008 |
| 27 | 27.630 | MM | 0.2266 | 2549.55737 | 187.50331 | 2.73678 |
| Totals : | | | | 9.31591e4 | 6835.41684 | |

===========================================================================

*** End of Report ***
