# Supplementary material for: Biostimulation of green microalgae Chlorella sorokiniana using nanoparticles of MgO, Ca10(PO4)6(OH)2, and ZnO for increasing biodiesel production
Source: Sci Rep. 2023 Nov 13;13:19730. doi: 10.1038/s41598-023-46790-w (PMC10643612; doi:10.1038/s41598-023-46790-w)
Supplement: Supplementary file 6 — Supplementary Information 6. [file 41598_2023_46790_MOESM6_ESM.pdf]

Data File C:\CHEM32\1\DATA\WEHDA\ALGEA_17000000.D
Sample Name:

===

Acq. Operator : support
Acq. Instrument : Instrument 1 Location : Vial 2
Injection Date : 12/22/2021 10:21:10 AM Inj : 1
Inj Volume : Manually
Acq. Method : C:\CHEM32\1\METHODS\FAME_NEW.M
Last changed : 10/4/2021 3:05:53 PM by support
Analysis Method : C:\CHEM32\1\METHODS\COOLING.M
Last changed : 9/12/2023 10:41:57 AM
(modified after loading)
Additional Info : Peak(s) manually integrated

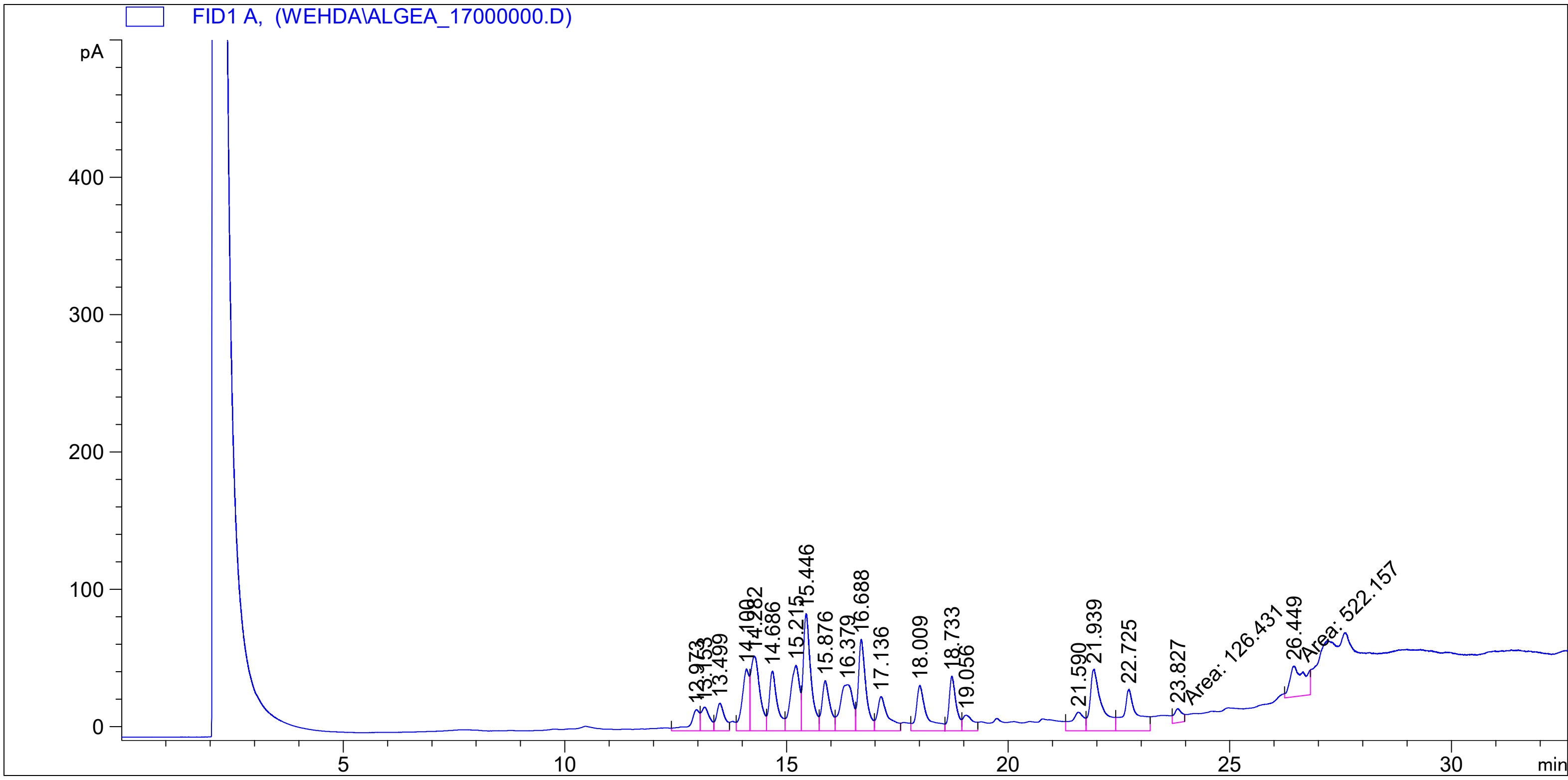

===

Area Percent Report

===

Sorted By : Signal
Multiplier: : 1.0000
Dilution: : 1.0000
Use Multiplier & Dilution Factor with ISTDs

Signal 1: FID1 A,

Peak #	RetTime [min]	Type	Width [min]	Area [pA*s]	Height [pA]	Area %
1	12.973	VV	0.1972	222.24457	15.32755	2.19771
2	13.153	VV	0.1886	228.21765	17.21880	2.25677
3	13.499	VV	0.1880	257.38727	19.88783	2.54522
4	14.100	VV	0.1560	485.82605	44.97427	4.80418
5	14.282	VV	0.1860	780.42731	54.20535	7.71740
6	14.686	VV	0.1788	548.11621	43.27328	5.42015
7	15.215	VV	0.1848	663.31433	47.52438	6.55930
8	15.446	VV	0.1730	1021.62036	85.14111	10.10248
9	15.876	VV	0.1819	460.42474	36.32275	4.55299

Peak #	RetTime [min]	Type	Width [min]	Area [pA*s]	Height [pA]	Area %
10	16.379	VV	0.2694	664.61145	33.51181	6.57213
11	16.688	VV	0.1783	787.06012	66.38735	7.78299
12	17.136	VV	0.2374	427.39932	24.75548	4.22642
13	18.009	VV	0.2330	545.13733	32.95892	5.39069
14	18.733	VV	0.1686	442.74631	39.54323	4.37818
15	19.056	VV	0.2190	189.05870	11.30952	1.86954
16	21.590	VV	0.2591	263.12653	13.39864	2.60197
17	21.939	VV	0.2394	818.53467	44.75619	8.09423
18	22.725	VV	0.2853	658.73065	30.10161	6.51398
19	23.827	MM	0.2078	126.43132	10.14247	1.25024
20	26.449	MM	0.3893	522.15686	22.35581	5.16344
Totals :				1.01126e4	693.09634	

===

*** End of Report ***
