# Supplementary material for: Biostimulation of green microalgae Chlorella sorokiniana using nanoparticles of MgO, Ca10(PO4)6(OH)2, and ZnO for increasing biodiesel production
Source: Sci Rep. 2023 Nov 13;13:19730. doi: 10.1038/s41598-023-46790-w (PMC10643612; doi:10.1038/s41598-023-46790-w)
Supplement: Supplementary file 7 — Supplementary Information 7. [file 41598_2023_46790_MOESM7_ESM.pdf]

Data File C:\CHEM32\1\DATA\WEHDA\ALGEA_18000000.D
Sample Name:

===

Acq. Operator : support
Acq. Instrument : Instrument 1 Location : Vial 2
Injection Date : 12/21/2021 1:29:19 PM Inj : 1
Inj Volume : Manually
Acq. Method : C:\CHEM32\1\METHODS\FAME_NEW.M
Last changed : 12/21/2021 1:15:36 PM by support
Analysis Method : C:\CHEM32\1\METHODS\COOLING.M
Last changed : 9/12/2023 10:41:57 AM
(modified after loading)
Additional Info : Peak(s) manually integrated

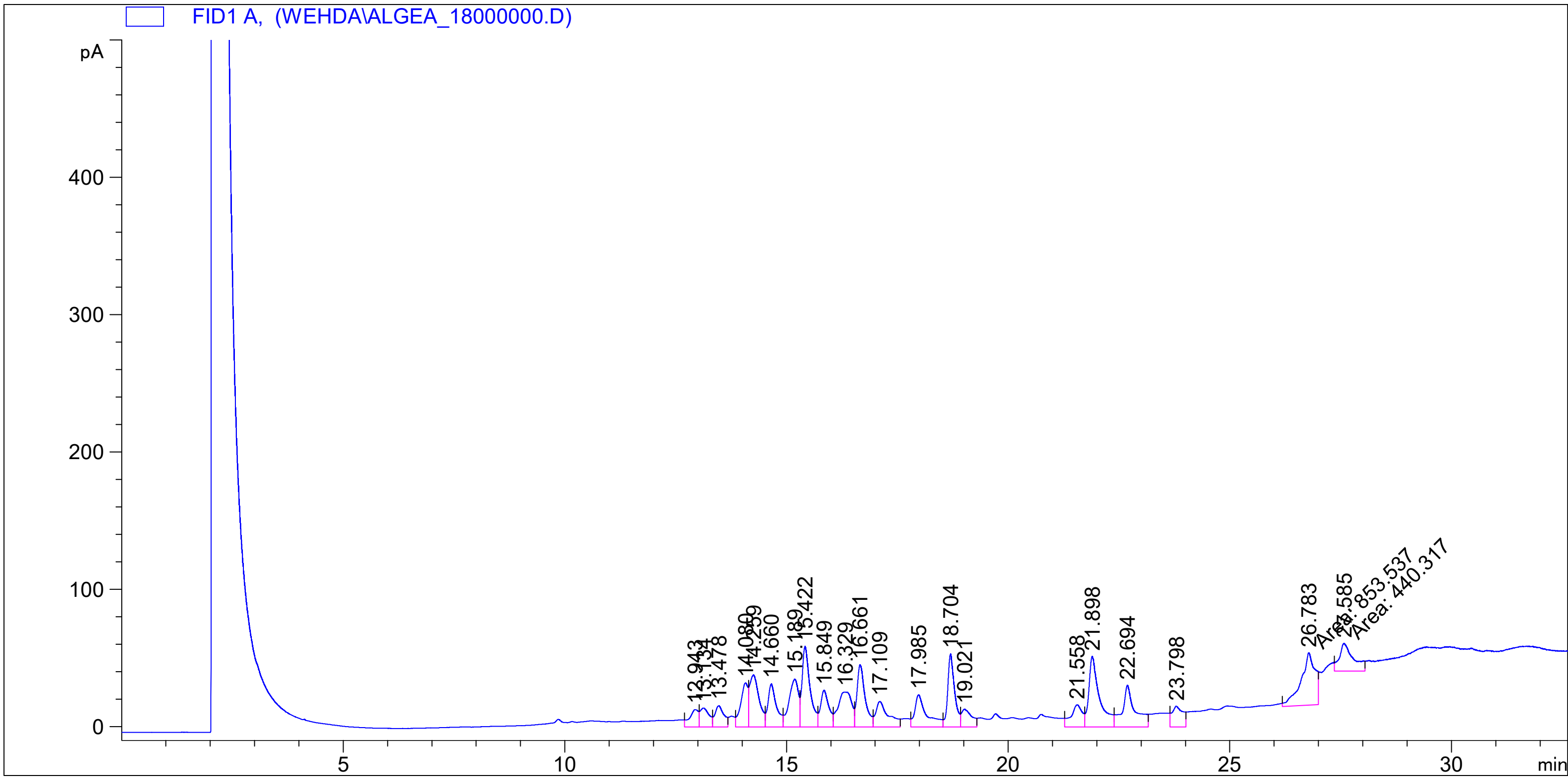

===

Area Percent Report

===

Sorted By : Signal
Multiplier: : 1.0000
Dilution: : 1.0000
Use Multiplier & Dilution Factor with ISTDs

Signal 1: FID1 A,

Peak #	RetTime [min]	Type	Width [min]	Area [pA*s]	Height [pA]	Area %
1	12.943	VV	0.1895	176.97131	12.78309	1.82808
2	13.134	VV	0.1949	201.96538	13.95069	2.08626
3	13.478	VV	0.2026	224.15057	15.59895	2.31543
4	14.080	VV	0.1576	362.97894	32.17773	3.74950
5	14.259	VV	0.2054	581.42456	38.02570	6.00599
6	14.660	VV	0.1976	424.58923	31.43400	4.38592
7	15.189	VV	0.1947	519.44830	35.08254	5.36579
8	15.422	VV	0.1766	748.11090	58.72233	7.72783
9	15.849	VV	0.1864	355.56628	26.87252	3.67292

Peak #	RetTime [min]	Type	Width [min]	Area [pA*s]	Height [pA]	Area %
10	16.329	VV	0.2566	549.24829	25.56932	5.67362
11	16.661	VV	0.1861	584.96307	45.46671	6.04254
12	17.109	VV	0.2758	376.80405	18.64177	3.89231
13	17.985	VV	0.2497	449.92422	23.46280	4.64762
14	18.704	VV	0.1503	576.35370	53.25555	5.95361
15	19.021	VV	0.2030	214.70087	13.11657	2.21781
16	21.558	VV	0.2459	288.24762	16.17768	2.97754
17	21.898	VV	0.2307	867.41626	51.45641	8.96023
18	22.694	VB	0.2701	620.06891	30.38072	6.40518
19	23.798	VV	0.2226	263.95468	15.20223	2.72660
20	26.783	MM	0.3735	853.53729	38.08677	8.81686
21	27.585	MM	0.3634	440.31689	20.19383	4.54838

Totals : 9680.74135 615.65791

==

*** End of Report ***
