# Supplementary material for: Biostimulation of green microalgae Chlorella sorokiniana using nanoparticles of MgO, Ca10(PO4)6(OH)2, and ZnO for increasing biodiesel production
Source: Sci Rep. 2023 Nov 13;13:19730. doi: 10.1038/s41598-023-46790-w (PMC10643612; doi:10.1038/s41598-023-46790-w)
Supplement: Supplementary file 8 — Supplementary Information 8. [file 41598_2023_46790_MOESM8_ESM.pdf]

Data File C:\CHEM32\1\DATA\WEHDA\ALGEA_7000000.D
Sample Name:

===

Acq. Operator : support
Acq. Instrument : Instrument 1 Location : Vial 2
Injection Date : 12/26/2021 1:01:38 PM Inj : 1
Inj Volume : Manually
Acq. Method : C:\CHEM32\1\METHODS\FAME_NEW.M
Last changed : 12/26/2021 12:48:18 PM by support
Analysis Method : C:\CHEM32\1\METHODS\COOLING.M
Last changed : 9/12/2023 10:41:57 AM
(modified after loading)
Additional Info : Peak(s) manually integrated

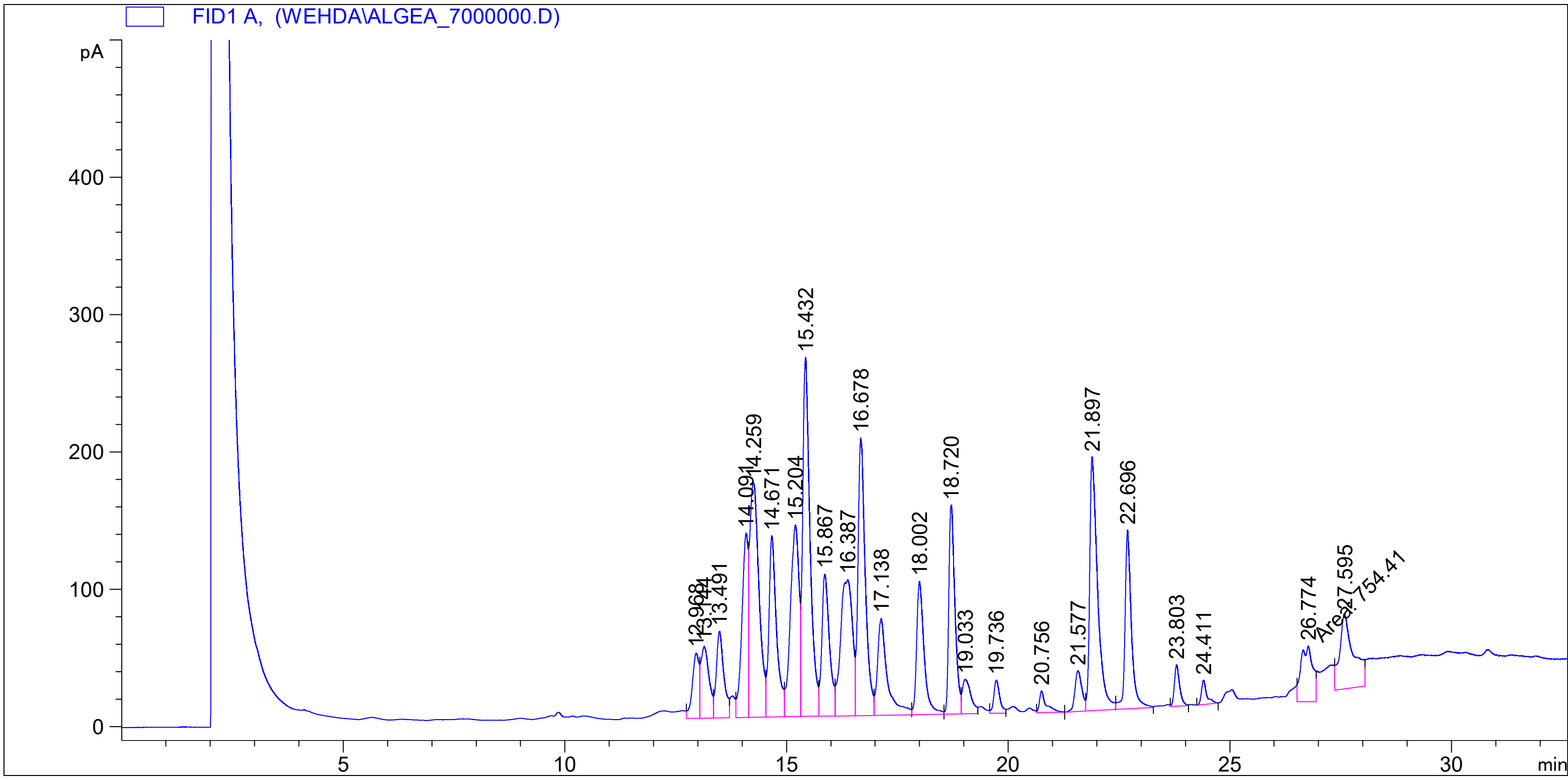

===

Area Percent Report

===

Sorted By : Signal
Multiplier: : 1.0000
Dilution: : 1.0000
Use Multiplier & Dilution Factor with ISTDs

Signal 1: FID1 A,

Peak #	RetTime [min]	Type	Width [min]	Area [pA*s]	Height [pA]	Area %
1	12.968	VV	0.1488	500.47781	47.55411	1.76048
2	13.144	VV	0.1668	680.12848	52.25837	2.39243
3	13.491	VV	0.1729	745.16553	63.03601	2.62120
4	14.091	VV	0.1401	1288.43408	134.07005	4.53221
5	14.259	VV	0.2146	2544.59106	170.06522	8.95088
6	14.671	VV	0.1731	1593.24695	131.75052	5.60442
7	15.204	VV	0.1890	1875.32983	139.33495	6.59668
8	15.432	VV	0.1622	3050.02344	261.22775	10.72879
9	15.867	VV	0.1643	1240.67407	103.19297	4.36421

Peak #	RetTime [min]	Type	Width [min]	Area [pA*s]	Height [pA]	Area %
10	16.387	VV	0.2551	1860.22998	99.04613	6.54356
11	16.678	VV	0.1712	2338.97266	201.78070	8.22759
12	17.138	VV	0.2171	1090.22742	70.21862	3.83499
13	18.002	VV	0.1714	1140.53015	96.83118	4.01194
14	18.720	VV	0.1452	1472.60205	151.76701	5.18004
15	19.033	VV	0.1801	339.88123	25.08389	1.19557
16	19.736	VV	0.1424	227.69131	24.05174	0.80093
17	20.756	VV	0.1485	164.39693	15.66300	0.57828
18	21.577	VV	0.1622	324.13641	29.49726	1.14018
19	21.897	VV	0.1694	2302.63843	184.73758	8.09978
20	22.696	VV	0.1497	1310.62524	129.92685	4.61027
21	23.803	VV	0.1163	248.54587	30.10664	0.87429
22	24.411	VV	0.1230	146.66188	17.64271	0.51590
23	26.774	MM	0.3101	754.41016	40.54241	2.65372
24	27.595	VV	0.2896	1188.78467	53.83953	4.18168

Totals : 2.84284e4 2273.22520

*** End of Report ***
