# Supplementary material for: Biostimulation of green microalgae Chlorella sorokiniana using nanoparticles of MgO, Ca10(PO4)6(OH)2, and ZnO for increasing biodiesel production
Source: Sci Rep. 2023 Nov 13;13:19730. doi: 10.1038/s41598-023-46790-w (PMC10643612; doi:10.1038/s41598-023-46790-w)
Supplement: Supplementary file 9 — Supplementary Information 9. [file 41598_2023_46790_MOESM9_ESM.pdf]

Data File C:\CHEM32\1\DATA\WEHDA\ALGEA_8000000.D
Sample Name:

===

Acq. Operator : support
Acq. Instrument : Instrument 1 Location : Vial 2
Injection Date : 12/27/2021 10:00:48 AM Inj : 1
Inj Volume : Manually
Acq. Method : C:\CHEM32\1\METHODS\FAME_NEW.M
Last changed : 10/4/2021 3:05:53 PM by support
Analysis Method : C:\CHEM32\1\METHODS\COOLING.M
Last changed : 9/12/2023 10:41:57 AM
(modified after loading)
Additional Info : Peak(s) manually integrated

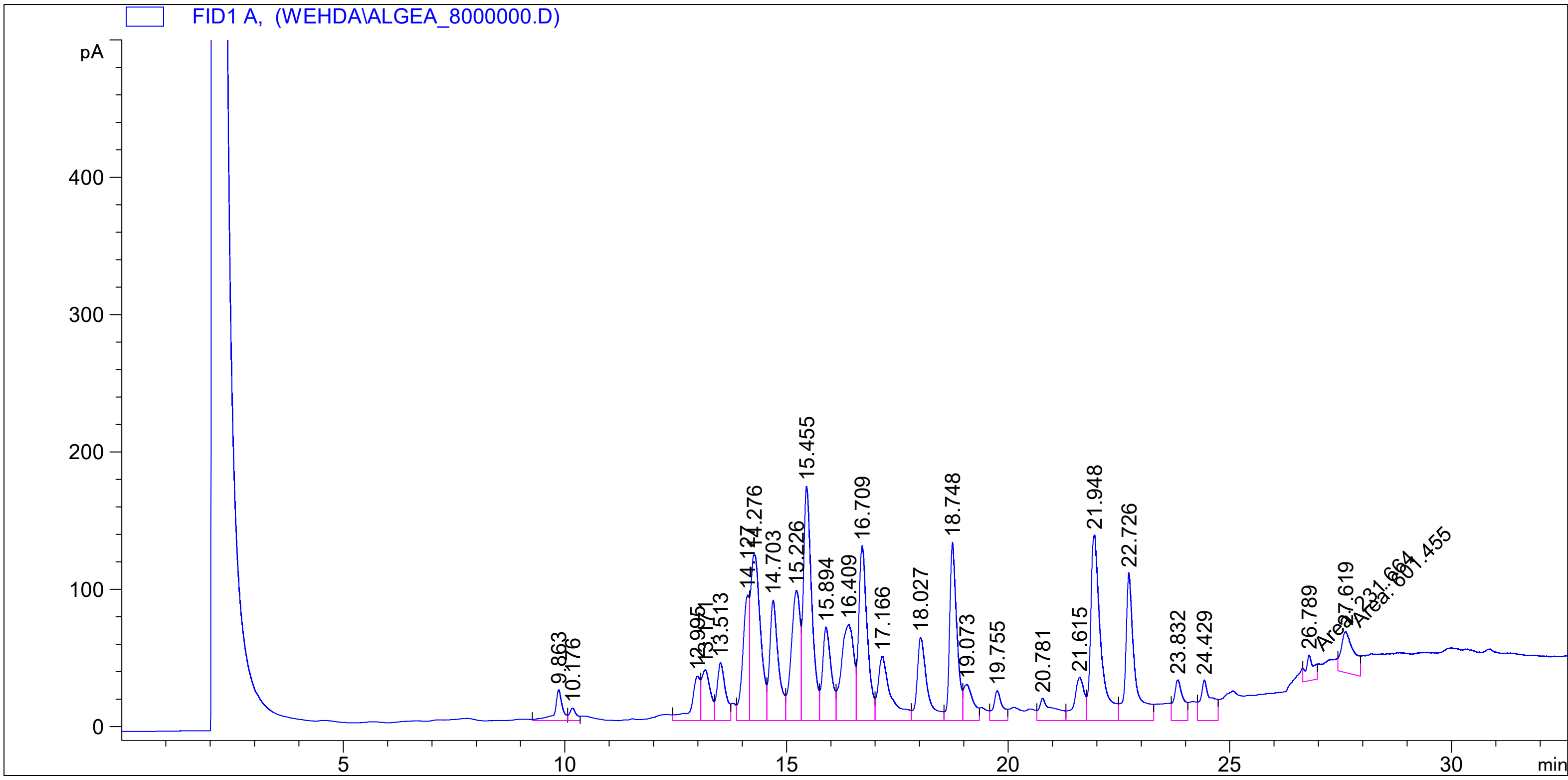

===

Area Percent Report

===

Sorted By : Signal
Multiplier: : 1.0000
Dilution: : 1.0000
Use Multiplier & Dilution Factor with ISTDs

Signal 1: FID1 A,

Peak #	RetTime [min]	Type	Width [min]	Area [pA*s]	Height [pA]	Area %
1	9.863	VV	0.1717	271.86090	22.38548	1.13268
2	10.176	VV	0.1529	97.31360	9.23724	0.40545
3	12.995	VV	0.1996	453.50244	32.54689	1.88946
4	13.171	VV	0.1924	513.25763	36.87715	2.13843
5	13.513	VV	0.1815	563.66730	42.27814	2.34845
6	14.127	VV	0.1458	899.67242	91.40909	3.74838
7	14.276	VV	0.2250	1911.41064	120.56954	7.96366
8	14.703	VV	0.1997	1209.91040	87.82352	5.04095
9	15.226	VV	0.2015	1326.17761	94.63078	5.52536

Peak #	RetTime [min]	Type	Width [min]	Area [pA*s]	Height [pA]	Area %
10	15.455	VV	0.1911	2265.91211	170.46346	9.44065
11	15.894	VV	0.2104	946.24896	67.97308	3.94243
12	16.409	VV	0.2414	1354.17981	70.10195	5.64203
13	16.709	VV	0.1991	1733.64783	127.14935	7.22303
14	17.166	VV	0.2729	959.37775	46.84290	3.99713
15	18.027	VV	0.2122	968.32983	60.67583	4.03443
16	18.748	VV	0.1703	1470.58044	129.65837	6.12700
17	19.073	VV	0.1955	407.50256	26.49207	1.69781
18	19.755	VV	0.1958	305.47903	21.75113	1.27274
19	20.781	VV	0.3036	382.55194	16.19471	1.59386
20	21.615	VV	0.2216	497.21198	31.59878	2.07157
21	21.948	VV	0.2169	2140.55591	135.05618	8.91837
22	22.726	VV	0.2023	1531.70825	107.47498	6.38168
23	23.832	VV	0.1997	435.03256	29.55627	1.81251
24	24.429	VV	0.2335	523.44373	29.43283	2.18087
25	26.789	MM	0.2084	231.66399	18.52468	0.96520
26	27.619	MM	0.3349	601.45520	29.93140	2.50589
Totals :				2.40017e4	1656.63580	

==

*** End of Report ***
