# Supplementary material for: Biostimulation of green microalgae Chlorella sorokiniana using nanoparticles of MgO, Ca10(PO4)6(OH)2, and ZnO for increasing biodiesel production
Source: Sci Rep. 2023 Nov 13;13:19730. doi: 10.1038/s41598-023-46790-w (PMC10643612; doi:10.1038/s41598-023-46790-w)
Supplement: Supplementary file 10 — Supplementary Information 10. [file 41598_2023_46790_MOESM10_ESM.pdf]

===

Acq. Operator : support
Acq. Instrument : Instrument 1 Location : Vial 2
Injection Date : 12/21/2021 10:42:29 AM Inj : 1
Inj Volume : Manually
Acq. Method : C:\CHEM32\1\METHODS\FAME_NEW.M
Last changed : 10/4/2021 3:05:53 PM by support
Analysis Method : C:\CHEM32\1\METHODS\COOLING.M
Last changed : 9/12/2023 10:41:57 AM
(modified after loading)
Additional Info : Peak(s) manually integrated

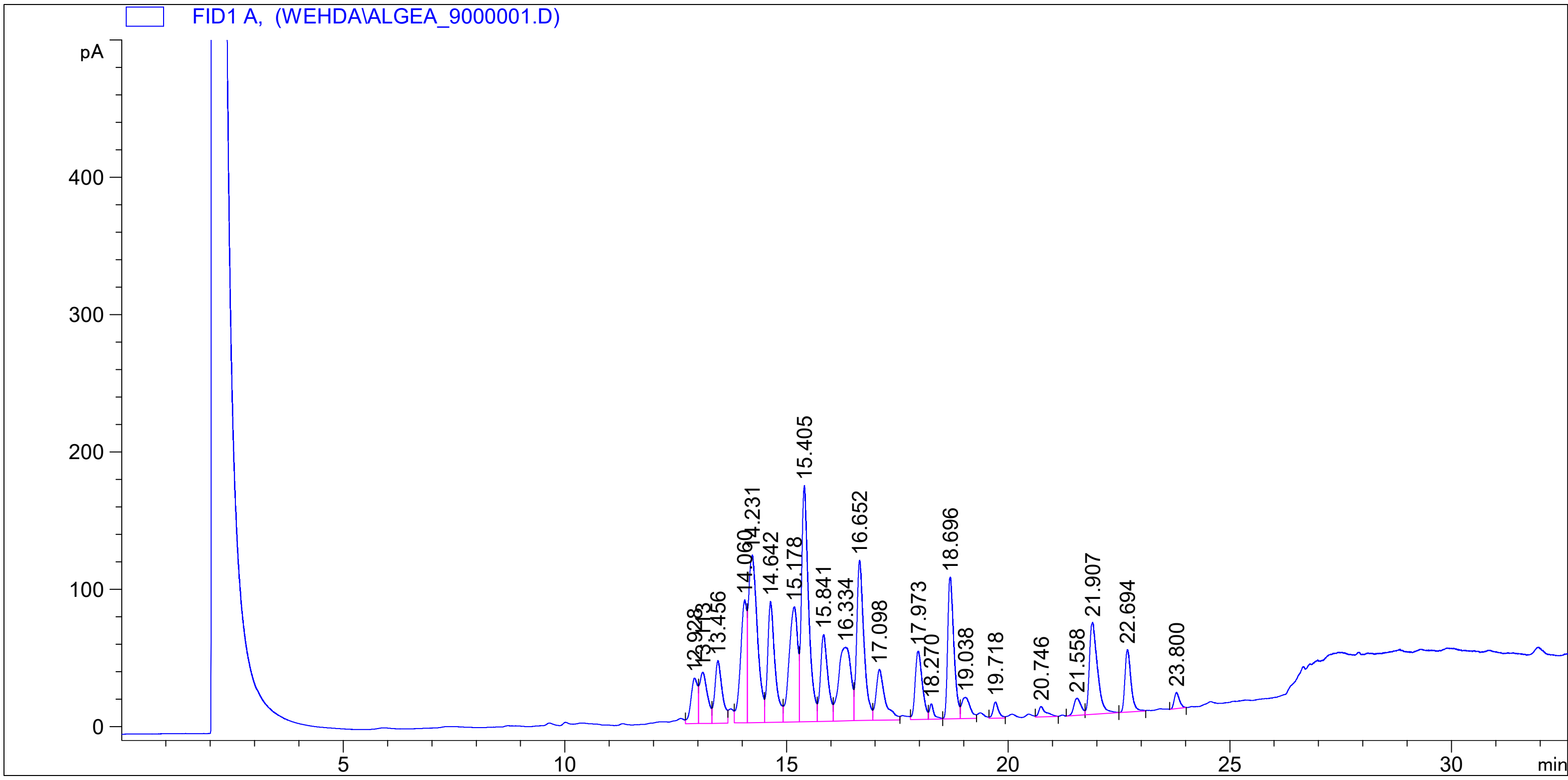

===

Area Percent Report

===

Sorted By : Signal
Multiplier: : 1.0000
Dilution: : 1.0000
Use Multiplier & Dilution Factor with ISTDs

Signal 1: FID1 A,

Peak #	RetTime [min]	Type	Width [min]	Area [pA*s]	Height [pA]	Area %
1	12.928	VV	0.1452	343.83450	33.08684	2.25685
2	13.113	VV	0.1793	471.89307	37.12599	3.09740
3	13.456	VV	0.1699	533.91028	45.49656	3.50447
4	14.060	VV	0.1444	861.05444	89.37432	5.65177
5	14.231	VV	0.1892	1736.07190	121.88377	11.39519
6	14.642	VV	0.1708	1029.89233	87.81409	6.75998
7	15.178	VV	0.2006	1122.30225	83.60970	7.36654
8	15.405	VV	0.1602	1945.44373	171.67203	12.76946
9	15.841	VV	0.1689	738.65540	62.96481	4.84837

Peak #	RetTime [min]	Type	Width [min]	Area [pA*s]	Height [pA]	Area %
10	16.334	VV	0.2427	1038.27454	53.43253	6.81500
11	16.652	VV	0.1668	1327.08936	116.60019	8.71072
12	17.098	VV	0.1975	522.66650	36.83972	3.43067
13	17.973	VV	0.1714	559.67023	49.67516	3.67355
14	18.270	VV	0.1033	79.82664	11.17528	0.52396
15	18.696	VV	0.1403	1008.91644	103.02425	6.62230
16	19.038	VV	0.1668	208.42679	15.50547	1.36807
17	19.718	VV	0.1257	99.84440	11.67906	0.65536
18	20.746	VV	0.1573	85.32317	7.58028	0.56004
19	21.558	VV	0.1721	141.97165	12.53893	0.93187
20	21.907	VV	0.1787	851.33295	66.77905	5.58796
21	22.694	VV	0.1428	431.65002	45.46194	2.83325
22	23.800	VV	0.1303	97.07974	11.75585	0.63721
Totals :				1.52351e4	1275.07581	

===

*** End of Report ***
