# Supplementary material for: Biostimulation of green microalgae Chlorella sorokiniana using nanoparticles of MgO, Ca10(PO4)6(OH)2, and ZnO for increasing biodiesel production
Source: Sci Rep. 2023 Nov 13;13:19730. doi: 10.1038/s41598-023-46790-w (PMC10643612; doi:10.1038/s41598-023-46790-w)
Supplement: Supplementary file 11 — Supplementary Information 11. [file 41598_2023_46790_MOESM11_ESM.pdf]

Data File C:\CHEM32\1\DATA\STANDARD\37FATTYACIDS001.D
Sample Name:

===

Acq. Operator : support
Acq. Instrument : Instrument 1 Location : Vial 2
Injection Date : 7/5/2021 12:40:32 PM Inj : 1
Inj Volume : Manually
Acq. Method : C:\CHEM32\1\METHODS\FAME_LONG.M
Last changed : 7/5/2021 12:26:40 PM by support
Analysis Method : C:\CHEM32\1\METHODS\COOLING.M
Last changed : 9/12/2023 11:01:07 AM
(modified after loading)
Additional Info : Peak(s) manually integrated

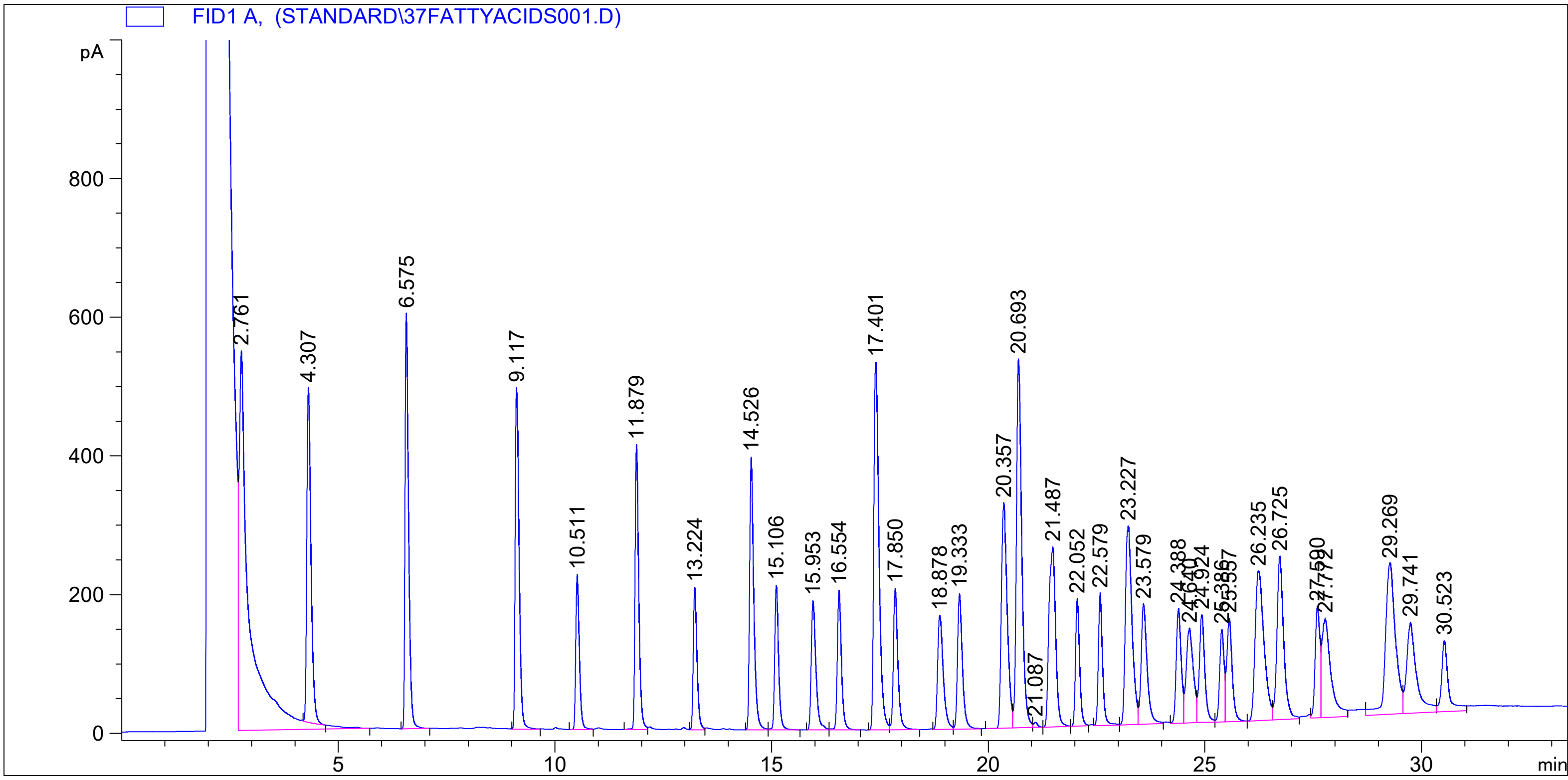

===

Area Percent Report

===

Sorted By : Signal
Multiplier: : 1.0000
Dilution: : 1.0000
Use Multiplier & Dilution Factor with ISTDs

Signal 1: FID1 A,

Peak #	RetTime [min]	Type	Width [min]	Area [pA*s]	Height [pA]	Area %
1	2.761	VB S	0.2850	9370.84473	547.19574	11.83340
2	4.307	VB X	0.1033	3278.35596	482.13412	4.13987
3	6.575	VB	0.0857	3421.74829	598.43005	4.32095
4	9.117	VV	0.0869	2862.37061	491.84564	3.61457
5	10.511	VV	0.0893	1286.12354	222.99466	1.62410
6	11.879	BV	0.0909	2458.24414	410.13120	3.10424
7	13.224	VV	0.0891	1213.10083	204.74055	1.53189
8	14.526	BV	0.1020	2626.14648	393.03464	3.31627
9	15.106	VV	0.0941	1302.45361	207.87012	1.64472

Peak #	RetTime [min]	Type	Width [min]	Area [pA*s]	Height [pA]	Area %
10	15.953	VV	0.1120	1401.92688	185.85722	1.77034
11	16.554	VB	0.1027	1339.05798	201.00070	1.69095
12	17.401	BV	0.1204	4376.52490	529.85516	5.52663
13	17.850	VB	0.1063	1469.52905	203.35640	1.85571
14	18.878	VV	0.1398	1483.35400	163.62578	1.87316
15	19.333	VV	0.1160	1520.85889	195.05183	1.92052
16	20.357	VV	0.1374	3040.29395	324.19830	3.83925
17	20.693	VV	0.1265	4573.84912	530.89368	5.77581
18	21.087	VV	0.0949	46.72705	7.27572	0.05901
19	21.487	VV	0.1508	2770.62915	259.05560	3.49872
20	22.052	VV	0.1008	1209.40735	183.67216	1.52723
21	22.579	VV	0.1041	1275.14636	190.39635	1.61024
22	23.227	VV	0.1548	3058.24805	285.82394	3.86192
23	23.579	VV	0.1446	1667.18005	172.78424	2.10530
24	24.388	VV	0.1241	1330.17041	164.89963	1.67973
25	24.640	VV	0.1419	1542.66785	136.43861	1.94806
26	24.924	VV	0.1262	1360.36316	155.23529	1.71785
27	25.386	VV	0.1125	962.41364	132.91701	1.21533
28	25.557	VV	0.1213	1288.92603	148.39041	1.62764
29	26.235	VV	0.2006	3071.96216	215.14674	3.87924
30	26.725	VV	0.1564	2547.22900	235.18666	3.21661
31	27.590	VV	0.1163	1260.15991	159.34576	1.59132
32	27.772	VV	0.1861	2024.68970	142.18942	2.55676
33	29.269	VV	0.2088	3543.40039	216.66408	4.47457
34	29.741	VV	0.2171	2073.89868	130.69083	2.61890
35	30.523	VV	0.1557	1131.76343	101.79409	1.42918
Totals :				7.91898e4	8930.12236	

===

*** End of Report ***
