# Supplementary material for: Biostimulation of green microalgae Chlorella sorokiniana using nanoparticles of MgO, Ca10(PO4)6(OH)2, and ZnO for increasing biodiesel production
Source: Sci Rep. 2023 Nov 13;13:19730. doi: 10.1038/s41598-023-46790-w (PMC10643612; doi:10.1038/s41598-023-46790-w)
Supplement: Supplementary file 12 — Supplementary Information 12. [file 41598_2023_46790_MOESM12_ESM.pdf]

Data File C:\CHEM32\1\DATA\WEHDA\ALGEA_4000000.D
Sample Name:

===

Acq. Operator : support
Acq. Instrument : Instrument 1 Location : Vial 2
Injection Date : 12/27/2021 12:49:07 PM Inj : 1
Inj Volume : Manually
Acq. Method : C:\CHEM32\1\METHODS\FAME_NEW.M
Last changed : 12/27/2021 12:32:47 PM by support
Analysis Method : C:\CHEM32\1\METHODS\COOLING.M
Last changed : 9/12/2023 10:41:57 AM
(modified after loading)
Additional Info : Peak(s) manually integrated

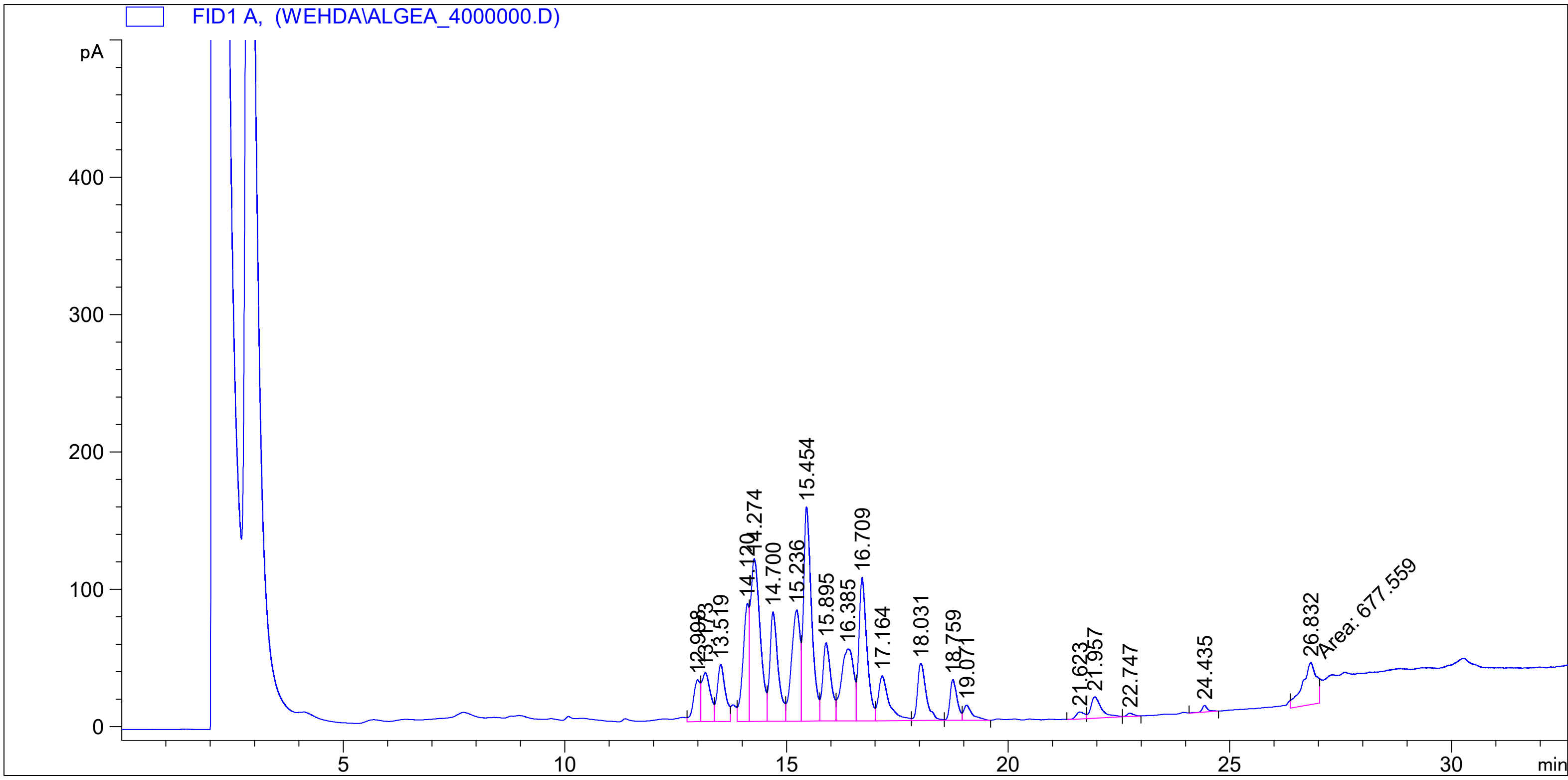

===

Area Percent Report

===

Sorted By : Signal
Multiplier: : 1.0000
Dilution: : 1.0000
Use Multiplier & Dilution Factor with ISTDs

Signal 1: FID1 A,

Peak #	RetTime [min]	Type	Width [min]	Area [pA*s]	Height [pA]	Area %
1	12.998	VV	0.1523	317.38690	30.52598	2.29502
2	13.173	VV	0.1964	494.34198	35.72662	3.57458
3	13.519	VV	0.1851	530.34735	41.50436	3.83493
4	14.120	VV	0.1317	786.07147	85.91594	5.68407
5	14.274	VV	0.2103	1818.25830	118.30070	13.14779
6	14.700	VV	0.1943	1039.38867	79.63449	7.51580
7	15.236	VV	0.1901	1091.70508	81.06187	7.89410
8	15.454	VV	0.1761	1990.86035	155.77371	14.39588
9	15.895	VV	0.1817	747.49579	56.75684	5.40513

Peak #	RetTime [min]	Type	Width [min]	Area [pA*s]	Height [pA]	Area %
10	16.385	VV	0.2379	1021.13806	52.27387	7.38383
11	16.709	VV	0.1792	1331.20532	104.08221	9.62592
12	17.164	VV	0.2265	550.90472	32.71587	3.98358
13	18.031	VV	0.1932	565.27863	41.41297	4.08752
14	18.759	VV	0.1531	327.51883	29.60403	2.36828
15	19.071	VV	0.1904	150.95012	11.04773	1.09152
16	21.623	VV	0.1714	64.00295	4.96636	0.46280
17	21.957	VV	0.2258	258.31586	15.47379	1.86788
18	22.747	VV	0.1464	25.06842	2.36931	0.18127
19	24.435	VV	0.1241	41.58263	4.79635	0.30068
20	26.832	MM	0.3676	677.55859	30.71742	4.89941
Totals :				1.38294e4	1014.66043	

===

*** End of Report ***
