# Supplementary material for: Biostimulation of green microalgae Chlorella sorokiniana using nanoparticles of MgO, Ca10(PO4)6(OH)2, and ZnO for increasing biodiesel production
Source: Sci Rep. 2023 Nov 13;13:19730. doi: 10.1038/s41598-023-46790-w (PMC10643612; doi:10.1038/s41598-023-46790-w)
Supplement: Supplementary file 13 — Supplementary Information 13. [file 41598_2023_46790_MOESM13_ESM.pdf]

===

Acq. Operator : support
Acq. Instrument : Instrument 1 Location : Vial 2
Injection Date : 12/15/2021 12:05:32 PM Inj : 1
Inj Volume : Manually
Acq. Method : C:\CHEM32\1\METHODS\FAME_NEW.M
Last changed : 12/15/2021 11:53:28 AM by support
Analysis Method : C:\CHEM32\1\METHODS\COOLING.M
Last changed : 9/12/2023 10:41:57 AM
(modified after loading)
Additional Info : Peak(s) manually integrated

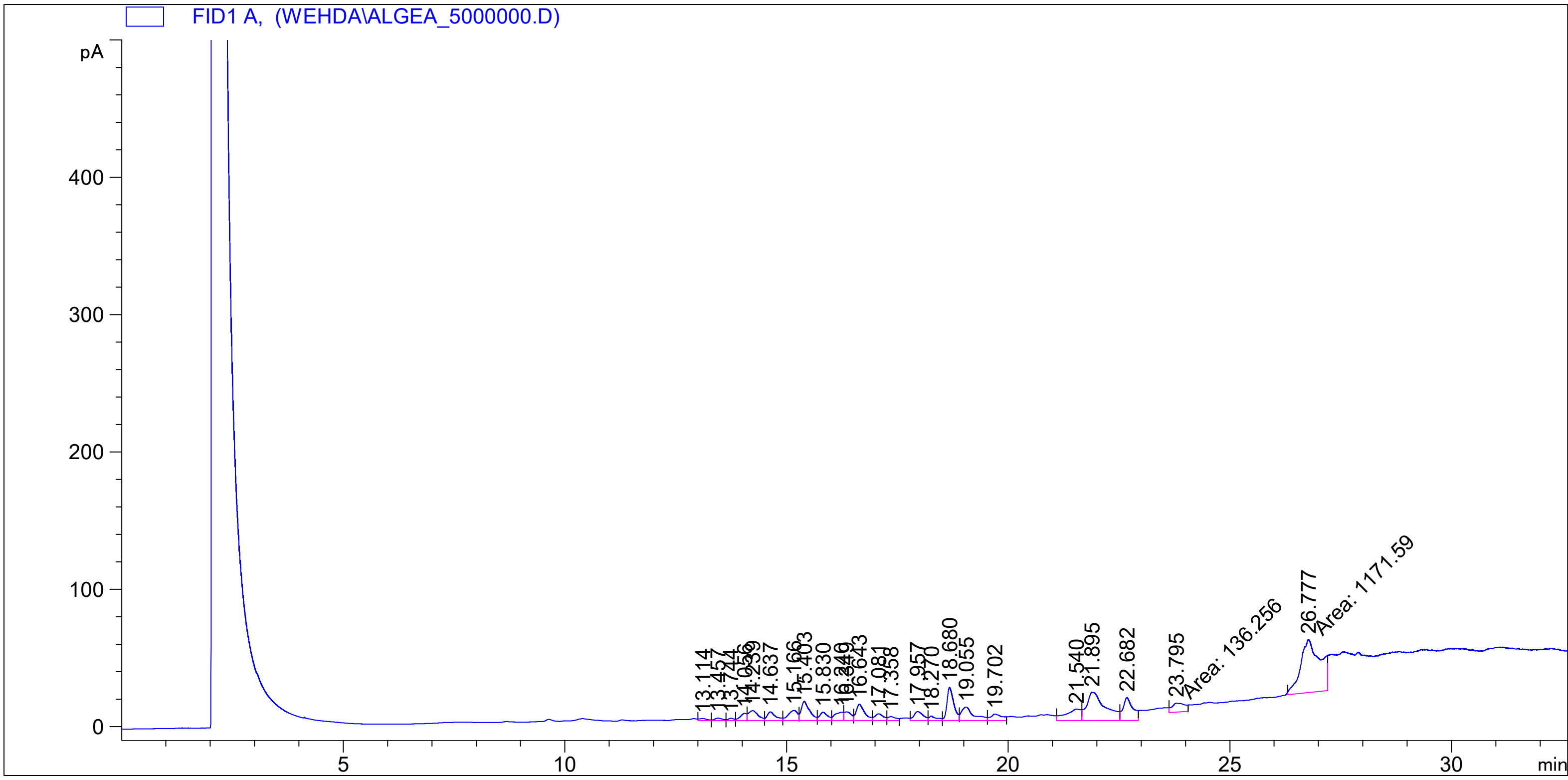

===

Area Percent Report

===

Sorted By : Signal
Multiplier: : 1.0000
Dilution: : 1.0000
Use Multiplier & Dilution Factor with ISTDs

Signal 1: FID1 A,

Peak #	RetTime [min]	Type	Width [min]	Area [pA*s]	Height [pA]	Area %
1	13.114	VV	0.1508	17.73637	1.45928	0.43236
2	13.457	VV	0.1497	20.78311	1.71144	0.50663
3	13.744	VV	0.1239	14.45152	1.62234	0.35228
4	14.056	VV	0.1336	50.90469	5.22597	1.24090
5	14.239	VV	0.2101	110.62244	7.16685	2.69664
6	14.637	VV	0.1912	84.62475	6.24106	2.06290
7	15.166	VV	0.1811	106.19751	7.28480	2.58878
8	15.403	VV	0.1842	184.64732	13.88298	4.50115
9	15.830	VV	0.1769	75.01341	5.99973	1.82860

Peak #	RetTime [min]	Type	Width [min]	Area [pA*s]	Height [pA]	Area %
10	16.240	VV	0.1654	79.37577	6.03773	1.93494
11	16.349	VV	0.1387	70.51478	6.19833	1.71894
12	16.643	VV	0.1894	157.92352	11.70753	3.84970
13	17.081	VV	0.1936	65.88927	4.93895	1.60618
14	17.358	VV	0.1629	36.19167	2.87405	0.88224
15	17.957	VV	0.1982	96.33612	6.45174	2.34838
16	18.270	VV	0.1612	39.34444	3.20632	0.95910
17	18.680	VV	0.1583	257.37158	24.15557	6.27394
18	19.055	VV	0.2574	188.80411	9.77215	4.60248
19	19.702	VV	0.2350	83.68251	4.67189	2.03993
20	21.540	VV	0.2901	196.37665	8.23918	4.78707
21	21.895	VV	0.3527	598.19836	20.48220	14.58227
22	22.682	VV	0.2152	259.39615	16.70136	6.32330
23	23.795	MM	0.3439	136.25610	6.60270	3.32151
24	26.777	MM	0.5070	1171.58728	38.51635	28.55977

Totals : 4102.22943 221.15050

*** End of Report ***
