# Supplementary material for: Biostimulation of green microalgae Chlorella sorokiniana using nanoparticles of MgO, Ca10(PO4)6(OH)2, and ZnO for increasing biodiesel production
Source: Sci Rep. 2023 Nov 13;13:19730. doi: 10.1038/s41598-023-46790-w (PMC10643612; doi:10.1038/s41598-023-46790-w)
Supplement: Supplementary file 14 — Supplementary Information 14. [file 41598_2023_46790_MOESM14_ESM.pdf]

Data File C:\CHEM32\1\DATA\WEHDA\ALGEA_6000000.D
Sample Name:

===

Acq. Operator : support
Acq. Instrument : Instrument 1 Location : Vial 2
Injection Date : 12/15/2021 12:44:53 PM Inj : 1
Inj Volume : Manually
Acq. Method : C:\CHEM32\1\METHODS\FAME_NEW.M
Last changed : 12/15/2021 12:38:15 PM by support
Analysis Method : C:\CHEM32\1\METHODS\COOLING.M
Last changed : 9/12/2023 10:41:57 AM
(modified after loading)
Additional Info : Peak(s) manually integrated

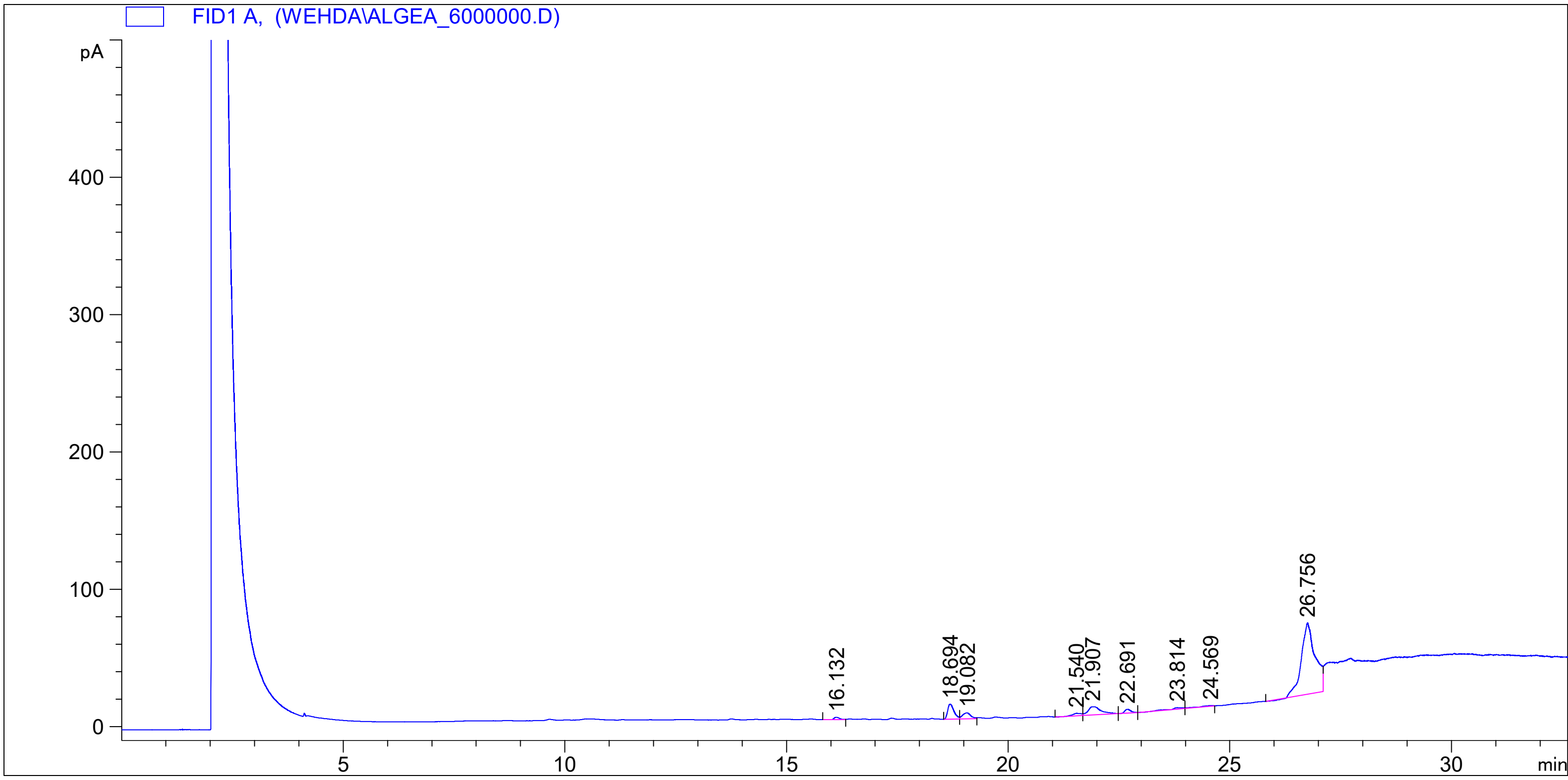

===

Area Percent Report

===

Sorted By : Signal
Multiplier: : 1.0000
Dilution: : 1.0000
Use Multiplier & Dilution Factor with ISTDs

Signal 1: FID1 A,

Peak #	RetTime [min]	Type	Width [min]	Area [pA*s]	Height [pA]	Area %
1	16.132	VV	0.1352	16.27175	1.78809	1.06409
2	18.694	BV	0.1453	109.45399	10.98200	7.15775
3	19.082	VV	0.1648	58.35354	4.36975	3.81603
4	21.540	VV	0.1677	23.95643	1.81861	1.56663
5	21.907	VV	0.2513	124.03609	6.04618	8.11135
6	22.691	VV	0.1260	27.47576	2.86202	1.79678
7	23.814	VV	0.2260	21.41806	1.14112	1.40064
8	24.569	VV	0.3066	15.78434	6.18714e-1	1.03222
9	26.756	VV	0.2693	1132.41699	51.75401	74.05450

Peak #	RetTime [min]	Type	Width [min]	Area [pA*s]	Height [pA]	Area %
Totals :				1529.16697	81.38048	

===

*** End of Report ***
